# Research on Object Detection in Cluttered Hospital Corridor Scenes with CSAWOA-YOLOv8

**DOI:** 10.3390/biomimetics11060431

**Published:** 2026-06-17

**Authors:** Tianye Luo, Jing Hu, Bangcheng Zhang, Xinming Zhang, Shaoming Luo

**Affiliations:** 1 School of Mechatronical Engineering, Changchun University of Science and Technology, Changchun 130022, China; 2024200136@mails.cust.edu.cn (T.L.); hujing@cust.edu.cn (J.H.); zxm@cust.edu.cn (X.Z.); 2 Changchun Institute of Technology, School Energy and Power Engineering, Changchun 130103, China; zhangbangcheng@ccut.edu.cn; 3 School of Mechatronic Engineering and Automation, Foshan University, Foshan 528225, China; 4 Precision Machining and Special Machining Innovation Team, Guangdong Education Department, Foshan 528225, China

**Keywords:** YOLOv8, CSAWOA, object detection, deep learning

## Abstract

Dynamic hospital corridor environments are characterized by complex corridor environments, diverse target-scale variations, frequent occlusions, and dense small-object distribution, posing significant challenges to the accuracy and efficiency of the existing methods on resource-constrained platforms. To effectively address these challenges, a high-precision framework CSAWOA (Cross Search Adaptive Whale Optimization Algorithm)-YOLOv8 (You Only Look Once version 8) model for complex medical environments was introduced in this work. By jointly modelling high-level semantic information and low-level cues such as texture and colour, the proposed model achieved a more discriminative and informative feature representation. The T-CBS (Transformer-Convolutional Bottleneck Structure) module, capable of extracting shallow-level features and integrating global contextual information to address target occlusion issues, was also proposed. Furthermore, the integration of the BiFormer module yielded an enhanced feature discriminability, improving small-target recognition while reducing sensitivity to background noise. The classification function was modified, effectively solving the problem of class imbalance in complex corridor environments. The combination of these two concepts achieved an effective balance of diversity in detection and convergence speed, leading to improved optimization performance and greater resistance to local-optimum stagnation. Meanwhile, an improved version of the WOA was developed, termed CSAWOA, enabling automatic hyperparameter optimization for the improved YOLOv8 model. From the experimental results, improvements of 4.9%, 6.1%, and 8.3% in mAP, precision, and recall, respectively, compared to YOLOv8 were demonstrated, while also exhibiting better generalization. Overall, the proposed method provides a reliable and efficient approach for object detection in complex hospital corridors, offering a valuable foundation for future research and real-world healthcare applications.

## 1. Introduction

The rapid evolution of AGV (Automated Guided Vehicle) technology has significantly enhanced automation capabilities across numerous sectors, with the healthcare industry benefiting from improved patient care efficiency, safety, and resource management [1]. AGVs have a wide range of applications in healthcare settings, including automated delivery robots and smart carts for streamlining logistics [2], patient transport vehicles that can navigate themselves and robotic helpers that aid medical personnel in improving the quality of their services [3]. This intelligent integration facilitates more efficient healthcare delivery by reducing waiting times, enhancing patient safety, optimizing medical staff utilization and lowering operational costs. AGVs, as a key representative of intelligent mobile service robots, are intended to achieve autonomous navigation from their starting position to the target location while avoiding collisions with obstacles. However, in dynamic and unpredictable environments, robots face severe limitations in perceiving local scenes, making it challenging to accurately assess road conditions. Consequently, the development of vision-based perception frameworks capable of accurately detecting and avoiding small obstacles in complex scenarios has emerged as a critical and prominent research focus in AGV autonomous driving systems [4].

Owing to their low cost, rich information content, and ease of integration, visual sensors—particularly cameras—have become a crucial component of AGV perception systems [5]. With the rapid development of deep learning techniques [6], camera-based environmental perception has achieved significant improvements in accuracy and robustness. These methods offer comparative advantages such as efficient data collection and relatively low computational requirements, making them well-suited for real-time, low-cost AGV autonomous driving systems [7]. Despite their widespread adoption, computer vision approaches continue to face challenges related to precise depth estimation, vulnerability to light variations, and reliable object recognition in occluded environments [8]. For instance, the SEF-NET approach presented by Tao Ye [9] achieved enhanced railroad obstacle detections by boosting speed and precision, especially for small objects in challenging circumstances. The framework synergistically combines modules for adaptive feature fusion, lightweight feature extraction, and reliable underlying feature extraction. In addition, the model effectively resolved the rail transportation obstacle detection issue. Wang et al. [10] introduced the MobileNet-YOLOv4 algorithm with the aim of improving the detection speed for autonomous driving applications. However, it resulted in reduced detection accuracy and persistent challenges in detecting small objects. By using multiple backbones to improve feature extraction and achieve excellent detection performance for tiny targets, Liu et al. [11] introduced a new dual backbone network detection method (DB-YOLOv5). Xu [12] et al. suggested the Attention YOLO method, which incorporates an attention mechanism based on YOLOv3 to increase detection accuracy for dense and obstructed targets. Nevertheless, its performance remains limited when detecting very small targets. Based on YOLOV4, Zhu et al. [13] generated feature maps at various scales by substituting residual concatenation for the original continuous convolution. Consequently, good detection results in situations with dense distributed environments were achieved. Zhou Dake et al. [14] proposed a dense human detection algorithm with a dual attention mechanism using RetinaNet as the basic framework, in order to improve pedestrian detection performance in scenes with a lot of occlusion. In the regression process, a spatial attention mechanism was incorporated, while in the classification branch, a channel attention sub-network was also included. However, the frame rate of this approach is relatively low, making real-time processing impossible; therefore, it cannot meet the application requirements of display scenarios. To deal with low identification accuracy of tiny and dense objects in urban street scenes, Yang [15] and colleagues suggested City-YOLO. Based on YOLOv5, City-YOLO provides an attention mechanism and a detection feature layer. In comparison to the previous model, an improvement of the mAP50 value by 5.8% was attained. This value was further enhanced by Yang [16] by adding a small object detection branch with a smaller detection size. Although the accuracy results were better with the upgraded YOLOv5, there were fewer target categories in this roadway dataset, and the performance decreased when applied in a hospital corridor context.

Intelligent optimization algorithms have been successfully applied to solve a variety of highly nonlinear and multimodal problems [17]. As a result, their application to deep neural networks has been gradually expanding in recent years. For example, Wang et al. proposed CPSOCNN, a novel variant of Particle Swarm Optimization, to optimize the hyperparameters of structurally determined CNNs [18]. The Fruit Fly Optimization Algorithm (FOA) was also used by Lee et al. to optimize the parameters of radial basis function neural networks (RBFNN) and generalized regression neural networks (GRNN), which reduced the need for human interaction in model creation and improved learning capabilities [19]. In particular, convolutional neural networks (CNNs), have achieved remarkable success in addressing a wide range of challenging tasks. However, their fine-tuning procedure is frequently intricate and time-consuming, with performance heavily reliant on hyperparameter selection. To address this issue, an improved Whale Optimization Algorithm (WOA) was presented in this work to optimize the learning rate of the proposed model, there by reducing the computational cost of reaching optimal performance.

A key limitation of the aforementioned feature extraction methods is their inability to capture cross-channel correlations, relying instead on simple concatenation of shallow and deep representations, which may limit the expressive power of the features. As a result, occluded and small targets remain difficult to detect, since features within each channel are isolated and inter-group information cannot be effectively captured from multiple inputs. This limitation prevents conventional detection frameworks from fully capturing complex scene characteristics, thereby reducing their detection accuracy and robustness. Therefore, effectively addressing detection challenges such as complex corridor environments, small object targets, and partial occlusions is essential for improving detection performance. Moreover, given that detection models are often deployed on edge computing devices, there is an urgent need to improve both accuracy and efficiency under such constraints. To tackle these challenges, a high-precision framework integrating intelligent algorithm optimization with object detection models was proposed in this work, with the goal of enhancing both detection speed and accuracy in hospital corridors with complex corridor environments.

### Contribution

**Figure 1:**
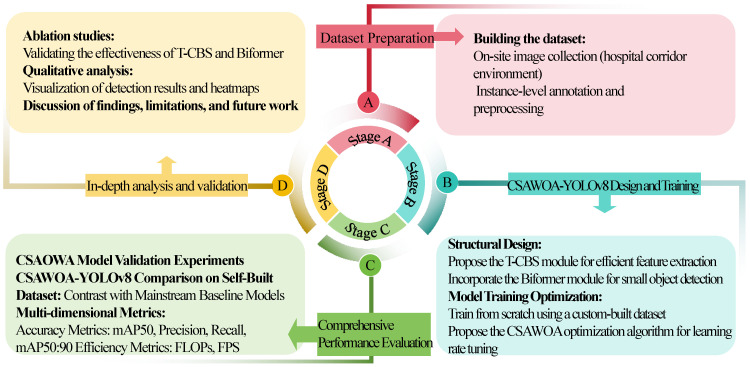
Overall workflow of this research. The research comprises four main phases: (**A**) dataset preparation, construction of a proprietary dataset; (**B**) CSAWOA-YOLOv8 framework design and training, focusing on optimizing the YOLOv8 model through the proposed new architecture and WOA; (**C**) comprehensive performance evaluation: benchmarking the proposed framework against baseline and state-of-the-art methods; (**D**) in-depth analysis and validation: conducting visual analyses of feature maps.

The dataset utilized in this work comprised hospital corridor scenes in a complex context. While state-of-the-art models have demonstrated impressive performance in object detection in normal images, the typical issues of wide variations in target scales, an abundance of small targets, and the influence of factors like environmental uncertainty necessitate further improvement of the current models. To overcome these limitations, this work focused on the recently developed YOLOv8 model and introduced targeted enhancements to improve its detection performance. Despite its substantial advances in detection accuracy and computational efficiency, YOLOv8 still faces limitations when deployed in challenging scenarios. Accordingly, an improved YOLOv8 framework designed to enhance detection accuracy, robustness, and computational efficiency in challenging hospital corridors scenarios was proposed in this work (Figure 1).

The main contribution of this work can be listed is as follows:

- An object detection model for hospital corridors in complex corridor environments based on the YOLOv8 structure was proposed. The approach completely integrates the benefits of both the Transformer and CNN structures. Interacting information between global and local features yielded the extraction of more effective features for the final target object in hospital corridors.
- To efficiently solve the class imbalance problem in complex corridor environments, the classification function of the detection head in the original YOLOv8 model was modified, and a Focal Loss function with adaptive adjustment of class weights based on iterative batches was proposed, which keeps the predicted outputs balanced among the classes in an effort to enhance the model’s performance.
- A new and improved version of the Whale Optimization Algorithm called CSAWOA was proposed to incorporate vertical and horizontal search mechanisms to enrich the diversity of populations, effectively improving the convergence speed and alleviating the exploration of local optima. The robustness and competitiveness of CSAWOA were well demonstrated through extensive experiments on IEEE CEC2017 and comparisons with 12 other algorithms. Targeting the YOLOv8 model’s hyperparameter optimization issue, a new optimization strategy was proposed to use the improved CSAWOA algorithm to make the model automatically select the optimal hyperparameters for the object detection problem in hospital corridors, so that the model achieves the optimal performance.
- An instance detection dataset tailored for complex hospital environments was constructed. The proposed CSAWOA-YOLOv8 framework demonstrated its effectiveness and generalization capabilities through validation on the self-built dataset.

The remainder of this work is organized as follows: in Section 2, the theoretical background underlying most of the methods employed in this work is introduced. The improved YOLOv8 model and CSAWOA algorithm, along with the entire optimized framework, are presented in Section 3. The experimental results and evaluation are detailed in Section 4. Then, in Section 5, the findings are compared with those of other similar studies, and the limitations are analyzed. Finally, the conclusions are presented in Section 6, where some directions for future work are also outlined.

## 2. Materials and Related Work

### 2.1. Dataset

**Figure 2:**
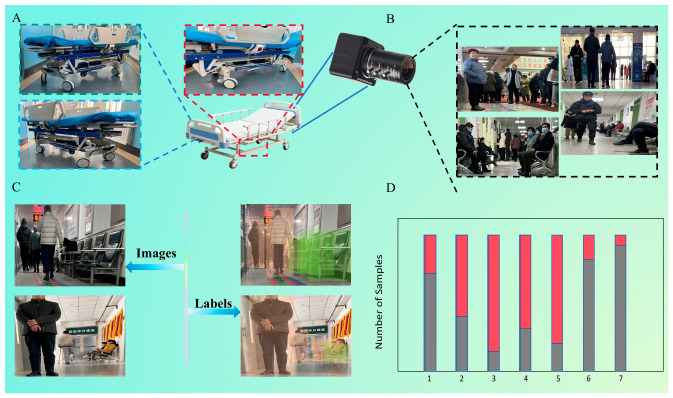
Related work on datasets. (**A**) Image acquisition equipment. (**B**) Examples of partial datasets. (**C**) Examples of labels in the self-built dataset. (**D**) Statistical analysis of positive and negative samples in the self-built dataset.

The dataset for this work consists of corridor scenes within complex hospital environments, captured multiple times from real-world settings at Jilin Central Hospital and The First Bethune Hospital of Jilin University. Data collection was conducted using cameras mounted on a custom-built intelligent transport vehicle, comprehensively capturing various actual hospital scenarios and potential obstacles, including people, trash bins, chairs, and other obstructions. The device configuration is shown in Figure 2A. The camera model is HBVCAM-12M2353 V11 (Shitong Yuntian Technology, Shenzhen, China), featuring 12-megapixel resolution and an 85° field of view.

Four thousand images taken in hospital hallways were chosen for this work, and the video data were processed for frame extraction at 15 frames per second. Six types of obstacles were marked: people, trash cans, seats, other cars, handbags, and fire hydrants. Every image was manually tagged and stored in the YOLO dataset format, and the annotation process makes use of the LabelImg tool(Python version 3.9). The data was subjected to image preprocessing procedures to avoid model overfitting. Due to the generally strong lighting conditions in hospitals, shadowed areas were present. The camera’s position relative to obstacles was fixed, containing irrelevant information for the study. Before being fed into the deep network, the images were cropped. Cropping was applied to reduce redundant information and focus on the most informative regions of the image. Although some obstacles may become partially occluded by the cropping operation, the retained image regions still preserve most of their relevant features. To establish our research foundation, 3500 raw images with a resolution of 4032 × 3024 pixels were selected and meticulously annotated. A representative example is shown in Figure 2B. The dataset was randomly divided into three groups, the training set, validation set, and test set, in an 8:1:1 ratio. As shown in Table 1. The data underwent image enhancing techniques, such as random cropping, Gaussian noise, flipping both horizontally and vertically, and scaling, in order to avoid model overfitting. After the enhancement process, the data volume increased to 5823 images. Figure 2C displays the gathered data samples. Table 2 shows the number of different categories in the dataset.

**Table 1:** Dataset summary table.

Data Split	Number of Images
Original images	3500
Augmented images	5823
Training set	4658
Validation set	582
Test set	583

**Table 2:** Distribution of dataset instances.

Category	Number of Instancese
Person	16,873
Trash bin	355
Chair	4718
Vehicle or cart	293
Handbag	8322
Fire hydrant	406

The dataset presents three major challenges. On the one hand, pedestrian obstacles are somewhat obscured by the comparatively fixed camera placements in the gathered pictures, which reduces the detection accuracy. Additionally, due to the influence of shooting distance, certain obstacles occupy only a small portion of the image, making them more susceptible to missed detections and leading lower recall rate. The complex environment of hospital corridors presents challenges distinct from common classification problems. The most significant issue is related to class imbalance. For example, the dataset was captured directly from a hospital environment. Due to the rich background information, it contains a large number of pedestrians among obstacles, while the number of other obstacles is relatively small. The significant variation in resolution creates an imbalance that poses considerable challenges during the model’s feature learning process. Severe sample imbalance reduces the recognition accuracy of significantly unbalanced characteristics, causing biassed parameter updates and consequently reducing recognition accuracy for underrepresented classes.

### 2.2. Focal Loss Function

The detection problem is addressed by Focal Loss (FL) [20], which lowers the loss weights of the well-detected samples. This allows the network to be trained on a sparse set of hard samples, preventing the classifier from being overwhelmed by easy negatives during the training process. As a result, the detrimental effects of class imbalance are mitigated. The challenging problem of positive and negative sample data imbalance may be effectively resolved by the fundamental concept of FL.

However, preliminary experimental results indicate that FL performs poorly when it comes to object detection tasks in complex corridor environments. Through careful analysis, it was found that this issue may be related to the FL mechanism, which inhibits the loss of well-classified samples, while in the scenario dealing with complex hospitals, it is more challenging to balance samples that fall in between two classifications. In this work, they were named hard-to-classify samples rather than hard examples. This effect can be attributed to the higher loss contributions of difficult samples, which naturally receive increased emphasis during training. However, these obfuscated yet low-loss samples receive limited emphasis during training, as their contribution to the optimization objective for the self-shooting distance problem is relatively small.

**Figure 3:**
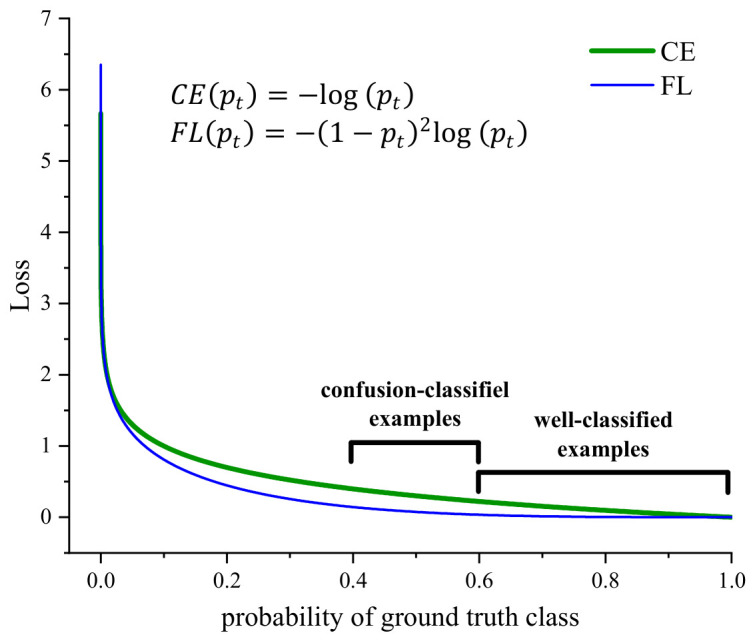
The FL and cross-entropy (CE) loss are shown in this graph.

A flatter loss function curve was produced when FL suppresses the confused samples’ loss values to a lower range of values than the CE [21] loss, as can be seen in Figure 3. This phenomenon might lessen the correlation between loss values and actual classification accuracy. Meanwhile, relatively limited research has been devoted to class imbalance in deep learning, with most studies concentrating on network design to enhance performance. However, class imbalance is common in real training datasets [22,23]. As deep learning technologies are applied to a broader range of real-world applications, the issue of class imbalance is expected to become increasingly significant. The poor performance of FL in the training outcomes of this work was caused by its insufficiency for class-balancing ability concerns.

Generally speaking, a significant disparity in the quantity of data among classes is referred to as class imbalance, which is a prevalent issue in real-world classification problems. The imbalance in class from two perspectives was systematically examined here. One form of class imbalance occurs at the data level, where the number of samples varies significantly among classes or between positive and negative examples. Resampling methods, including majority class undersampling and minority class oversampling, have been extensively adopted to alleviate the effects of class imbalance during model training [24,25,26]. The underlying data distribution is known to be affected by this method, which also raises the possibility of overfitting and more computational work. Classifier integration is an additional method of resampling the training data in which a distinct sample from the original dataset induces each classifier [27,28]. The dataset is expanded for model training using generative adversarial networks to better understand the dataset distribution [29,30]. In addition, other techniques such as iterative sampling [31] and small incremental corrections [32] have been adopted for the training of deep neural networks. An alternative perspective is to divide the training samples according to their learning difficulty, distinguishing between easy and hard examples. This concept forms the foundation of the FL mechanism. As a result, the loss of easy samples is suppressed, the network is concentrated on a small number of hard samples for training, and the samples’ classification difficulty is directly assessed based on the confidence scores in the predictions matching the positive categories in the labels. According to this perspective, the model’s training process entirely disregards the class information in the training data. However, it is strongly tied to the prediction outcomes, which forces the model to continue concentrating on the hard samples in order to improve its performance.

With outstanding performance, FL and its variations have been applied extensively in a variety of deep learning classification and detection problems since its proposal [33,34,35]. Therefore, in this work, an improved version of the FL function was incorporated into the proposed YOLOv8 framework, replacing the BCE classification function to better address the class imbalance problem and improve detection performance in complex hospital scenarios.

## 3. Method

To address issues in centralized data, the CSAWOA-YOLOv8 object detection framework for hospital corridors was proposed. First, the overall architecture for improving the YOLOv8 model was presented. Subsequently, the proposed module was introduced to address issues such as occlusion and small objects in the dataset, while also improving the FL function to take the role of the YOLOv8 model’s original classification function. To save time and resources during training and obtain optimal values for improving YOLOv8 hyperparameters, a new algorithm—CSAWOA—was proposed based on the WOA. Finally, the process of optimizing and improving the YOLOv8 model hyperparameters using CSAWOA was explained.

### 3.1. Improvement of YOLOv8 Model

**Figure 4:**
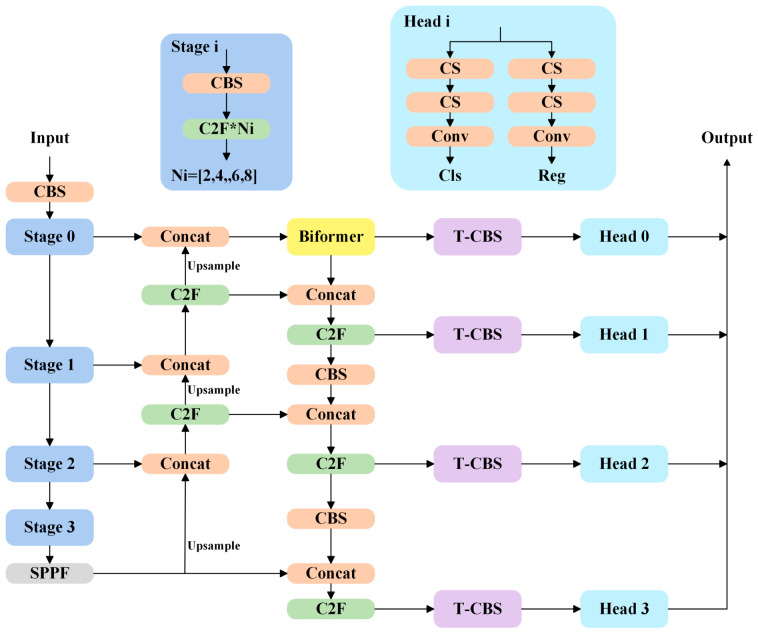
Improved YOLOv8 overall structure.

Because of its exceptional computing efficiency and very low number of parameters, the YOLO (You Only Look Once) family of models has emerged as one of the most popular methods in the field of object detection. Their computational efficiency makes them particularly attractive for deployment on automation systems with limited resources, facilitating reliable real-time on-site visual perception. It is important to note that, in the evaluation paradigm, the YOLO instance detection model differs significantly from conventional object detection methods. More crucially, correct localization, category identification for every target instance, and careful boundary delineation are needed throughout the detection phase in addition to exact pixel-level categorization. Considering the YOLO framework’s enormous potential and ongoing development in object detection tasks, the YOLOv8 [36] model was adopted in this work as its fundamental architecture. The proposed improved structure is illustrated in Figure 4.

#### 3.1.1. T-CBS Module

**Figure 5:**
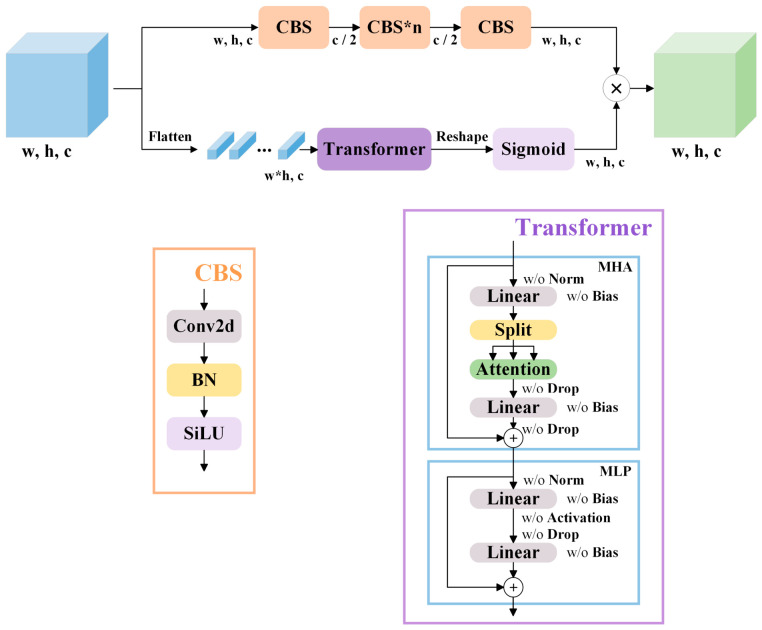
T-CBS structure diagram. The letters w, h, and c denote the width, height, and channel dimensions, respectively. The abbreviation w/o stands for ‘with or without.’ Norm, Drop, and Activation refer to Layer Normalization (LayerNorm), Dropout, and Gaussian Error Linear Unit (GeLU) activation, respectively. Bias indicates the bias term in the linear layer.

Due to the limitations of the limited shooting distance and the complex corridor environments, the samples of the dataset in this work present characteristics different from those of natural images, such as various sizes, great density, restricted features, and a complicated backdrop. Object detection in medical contexts benefits greatly from the ability of global context and extensive information to help detectors locate obstructed targets. However, due to the nature of convolutional processes, standard CNN-based detectors are significantly lacking in global interactions. A unique plug-and-play module named T-CBS was developed in this work with the goal of integrating local features with global contextual information in order to address this important issue. It is composed of two primary parts: a convolutional bottleneck block that extracts local features and supplies inductive bias to the other components, and a simplified Transformer layer that is in charge of capturing remote dependencies. Two reshaping procedures and a more basic Transformer variation were included in the simplified Transformer layer, as can be seen in Figure 5. Although Transformer-based models, such as ViT [37], have achieved remarkable success in computer vision, the computational characteristics and feature extraction mechanisms of conventional Transformers may not be ideally suited for the complex hospital setting. As demonstrated by the studies presented in Section 4, the original transformer is not a suitable form, at least for T-CBS. In this work, the simplified Transformer computes self-attention using single-head attention and eliminates LayerNorm [38], Dropout, and GeLU [39]. Multi-head attention and multi-layer perception (MLP) are its two main components. In order to process the 2D feature mapping, the mapping $X\in R^{H\times W\times C}$ is flattened to $X_{t\in }R^{(H\times W)\times C}$ and fed into the simplistic Transformer, where (H,W,C) denotes feature map resolution. Value, key, and query can be calculated as follows:

(1) $$Q,K,V=Split(Linear(X_{t}))$$

A technique called split divides a matrix into blocks along the channel dimension, whereas linear denotes one of the fully connected layers. If Q, K, V, and X are constant in size, the self-attention formula can be expressed as follows:

(2) $$Attention=SoftMax(QK^{T}/C^{0.5})V$$

The following expression represents the multi-head attention’s final output X_s for the remaining connections:

(3) $$X_{S}=Linear(Attention)+X_{t}$$

There are no GeLUs in the first layer of the MLP, which has two completely linked layers. The entire procedure can be characterized as follows, where X denotes the output of the MLP layer.

(4) $$X_{mlp}=Linear(Linear(X_{S}))+X_{S}$$

In conclusion, the output of the Xtran simplified Transformer layer can be expressed as follows:

(5) $$X_{tran}=Transformer(X)$$

The original mapping is replaced by a conventional attention mechanism that conditions the feature mapping after the convolutional layer. The features produced by the simplified Transformer layer and the convolutional bottleneck block are then fused using element-wise multiplication. This allows for the acquisition of each spatial location’s features while being guided by local information and global context. As a supplementary integration, T-CBS adds long-range dependencies for CNN and local centralization for Transformer. By integrating the T-CBS module, the enhanced model achieved superior performance while demonstrating improved generalization capability. In the end, T-CBS as a whole can be officially represented as follows where $X_{T-CBS}$ and ⊗ refer to the output of T-CBS and elemental multiplication, respectively.

(6) $$X_{T-CBS}=X_{conv}⊗X_{tran}$$

#### 3.1.2. BiFormer

**Figure 6:**
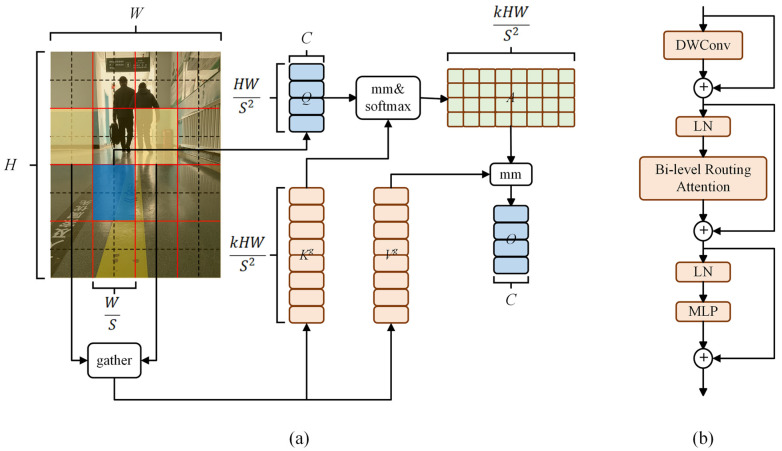
The structure diagram of BiFormer. (**a**) The bi-level routing attention structure. (**b**) Composition of the BiFormer block.

The coexistence of complex corridor environments information and small-target instances presents a substantial challenge for object detection models, often reducing their ability to effectively discriminate foreground objects from irrelevant scene information. To assist the detection model in putting less emphasis on the background information and focusing more on the important data in the input features, a dynamic sparse attention approach known as BiFormer [40] was introduced into the model’s network. Its structure is shown in Figure 6.

**Figure 7:**
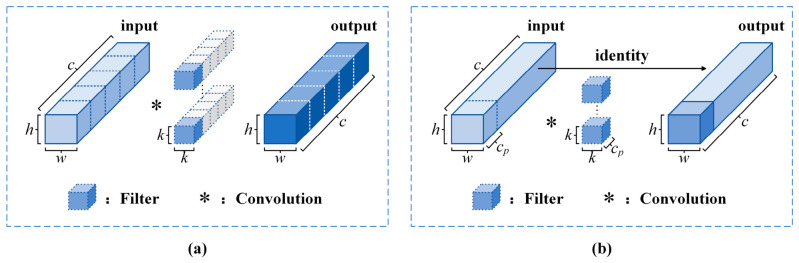
Comparison of DW convolution and PW convolution. (**a**) DWConv structure. (**b**) FasterNet block structure.

The inclusion of a detection scale in the model has emerged as an effective technique in recent object detection research, enabling better preservation of fine-grained features and significantly reducing missed detections of small targets [41]. Nevertheless, this method leads to an increase in the amount of computing and storage resources used and complicates the model structure. To solve this problem, DWConv has been improved to greatly increase the precision of the detection of tiny objects while cutting down on unnecessary resource utilization. DWConv is shown in Figure 7a. Due to computational resource constraints, the object detection task necessitates the search for models with low latency, high data throughput, and a simple structure. Some traditional lightweight networks, including MobileNet [42], ShuffleNet [43], and GhostNet [44], use group convolution or deep convolution to extract spatial information from images. By convolving the input image grouped by feature dimensions, deepthwise separable convolution preserves the consistency of feature dimensions and feature information while reducing the number of feature dimensions and the number of parameters. Nevertheless, the model’s computational speed does not necessarily increase when the model parameters are reduced. Consequently, researchers have proposed lightweight neural network blocks that leverage deep or group convolution to reduce computational complexity. In several instances, this approach has worsened delays rather than accelerating the model.

(7) $$FLOPs_{conv}=h\times w\times k^{2}\times c^{2}$$

The necessary FLOPs using regular convolution of size k×k are displayed in Equation (7) for input features of size h×w×c, where cindicates the quantity of input data channels.

To get the output channel features, Equation (8) for deep convolution was used to compute the FLOPs, after which the deep convolution kernel was applied across the input channel space.

(8) $$FLOPs_{conv}=h\times w\times k^{2}\times c$$

The well-liked deep convolution successfully lowers the model’s parameters. However, in practice, point-by-point convolution or other computing costs are frequently used after deep convolution to compensate for the accuracy loss that occurs after the convolution process. This raises latency and adds to the cost of memory access. Because of this, the FasterNet concept [45] and partial convolution (PConv) with the structure depicted in Figure 7b in place of depth separable convolution were utilized. Some continuous features in the input channel undergo a convolution operation by PConv, while the remaining features undergo constant mapping processing, which maintains the channel’s integrity. The formula for computing FLOPs for PConv is derived from this procedure.

(9) $$FLOPs_{pconv}=h\times w\times k^{2}\times c_{p}^{2}$$

By adjusting c_p to one-fourth of the input feature channel count c, the computational complexity of PConv is greatly reduced, with the resulting FLOPs amounting to only 6.25% of those associated with traditional convolution. This convolution efficiently extracts the image’s spatial feature information while lowering the amount of memory accesses and parameters. 8×, 16×, and 32× downsampled feature maps can be created by the stage sequence when the pre-processed image is fed into the backbone network as an input image with additional gradient routes through the C2F structure. In order to enhance coarse-grained object localization information and fine-grained object top-down information, these feature mappings were then routed to a framework for route aggregation that comprises both top-down and bottom-up paths. Lastly, the detection head predicts each mesh’s classes and bounding boxes using enhanced feature mapping. In complex contexts, a T-CBS structure is introduced between the detection header and the path aggregation structure to integrate local characteristics and global contextual information for target recognition in hospital hallways. Even though YOLOv8 is more accurate than other popular algorithms in detecting small objects, this paper’s dataset still has some omissions and false positives. To address this problem, an additional branch was added to detect small targets at stage 0 when the sensing field was smallest and the underlying information was largest. This path introduces a dynamic sparse attention technique to harvest information from extremely tiny target layers. The integration of this module enhances the model’s detection ability, enabling more effective attention to crucial target information. The loss function for classification was modified in the detection header, replacing the original BCE function with an improved Focal Loss function.

#### 3.1.3. Improve Focal Loss Function

The original FL treats all categories equally, ignoring the impact of differences in sample size between categories on loss weights. In the dataset used in this work, instances of the ‘Person’, ‘Chair’ and ‘Handbag’ categories constitute the majority, whilst instances of the remaining categories are few in number. Consequently, fewer positive samples were obtained during training, resulting in a lower contribution to the loss. This leads to insufficient updating of model parameters, thereby causing low accuracy for underrepresented categories. To address this issue, a class weighting factor [46] was incorporated into the loss function during training. This effectively resolves the class imbalance problem, thereby maintaining a balance in the prediction outputs across all classes and improving the model’s performance.

Currently, the median frequency balancing method [47] is commonly used to set the class weighting factor. This method originates from semantic segmentation, where the pixel serves as the statistical unit, and it ignores the dynamic changes in sample distribution during training. This concept was transferred here to the positive sample level in object detection and improved upon the method used in SegNet [48] by implementing adaptive adjustment based on iterative batches.

First, in the s-th iteration, the number of positive samples ncs assigned to class c by the allocator was counted. Let the total number of positive samples in this batch be $N_{pos}^{s}=\sum_{c\in C}n_{c}^{s}$. Then, the instantaneous frequency of class c can be described in Equation (10).

(10) $$f_{c}^{s^{\prime }}=\frac{sum(y_{c})}{h\times w\times bs}$$

Secondly, the category frequency fcs was calculated by weighting the intermediate value of the instantaneous frequency fcs’ at the current sth iteration and the frequency fcs−1 from the (s-1)th iteration; the calculation method is shown in Equation (11).

(11) $$f_{c}^{s}=\frac{f_{c}^{s^{\prime }}+(S-1)\times f_{c}^{s-1}}{S}$$

Consequently, the frequency fcs of category C was adaptively adjusted on an iterative batch basis, with adjustments occurring once per iterative batch, in order to achieve dynamic adaptive adjustment of the weights. $W_{c}$ refers to the weight coefficient assigned to class c, equal to the ratio of the median of all class frequencies at the current sth iteration to the frequency fcs of class c, calculated as follows.

(12) $$W_{c}=\frac{median(\{f_{c}^{s}|c\in C\})}{f_{c}^{s}+\varepsilon }$$

The median ( ) function denotes the median of the frequencies corresponding to the C categories. To avoid division by zero when fc is 0 due to the absence of a particular category in the image, ε was set to $10^{-4}$.

Minority classes with low frequencies yield large $W_{c}$ values, which are subsequently amplified in the loss function; $W_{c}$ was then normalized and modulated to obtain the per-sample category weights $\hat{Z}$.

(13) $$\hat{Z}=\frac{W_{c}}{\sum_{c\in C}\left(W_{c}\right)}\times \frac{\left(1+y_{c}+p_{t}\right)}{2}$$

where $y_{c}$ is the classification label for category c of the positive sample, and pt is the model’s predicted probability of the target category for that sample. The final L1 loss function is shown below.

(14) $$L_{1}=-\frac{1}{N}\sum_{i=1}^{N}\sum_{c=1}^{c}\hat{Z}(1-p_{t}{)}^{\gamma }log(p_{t})$$

where $L_{1}$ is a per-sample loss and is sensitive to class imbalance and the difficulty of samples. To provide a complementary training signal at the set level, a Dice term was introduced [49]. Unlike its definition in segmentation for pixel masks, here Dice is defined on the anchor point grid: for each class c, the predicted probability and the assigned target at all anchor points are treated as two vectors, and their soft overlap is measured as follows:

(15) $$Dice_{c}=\frac{2\sum_{i=1}^{N}P_{i,c}g_{i,c}+\varepsilon }{\sum_{i=1}^{N}p_{i,c}^{2}+\sum_{i=1}^{N}g_{i,c}^{2}+\varepsilon }$$

where $p_{i,c}$ is the model’s predicted probability that the i-th anchor point belongs to class c, $g_{i,c}$ denotes the aligned classification target for class c of the i-th anchor point provided by the TaskAlignedAssigner in YOLO, and *ε* refers to a smoothing term. Subsequently, the Dice scores for each class are averaged and log-transformed to obtain $L_{2}$.

(16) $$L_{2}=-log(mean\{dice|c\in C\})$$

The Dice term measures the overall consistency between the predicted class distribution and the assignment targets at the level of the entire anchor grid; it is insensitive to the absolute number of positive and negative samples and complements the per-sample $L_{1}$ loss, aiding in stable training under severe class imbalance.

The resulting $L_{1}$ and $L_{2}$ are weighted, where αϵ[0,1] serves as a weighting adjustment factor, yielding the final improved Focal Loss function.

(17) $$\begin{array}{l} Improved\;focal\;loss=\alpha L_{1}+(1-\alpha )L_{2}=\\ -\alpha \frac{1}{N}\sum_{i=1}^{N}\sum_{C=1}^{C}\hat{Z}{\left(1-p_{t}\right)}^{\gamma }log(p_{i})-(1-\alpha )log(\{meand_{c}|c\in C\}) \end{array}$$

Following this approach, at each iteration, weights $\hat{Z}$ are generated adaptively based on the number of positive samples in each class. This coordinates the classification loss constraints at both the sample and set levels, fully utilizing the distribution characteristics of positive samples across classes in the current training batch, thereby alleviating the issue of class imbalance.

### 3.2. CSAWOA

Training object detection models in hospital corridor environments is particularly challenging due to inherent difficulties in complex visual scenes, including strong background interference, imbalanced positive-to-negative sample ratios, and severe target occlusion. First, complex corridor environments imply the presence of substantial distracting information unrelated to the core task within the image, such as fluctuating lighting conditions and passing individuals. These disturbance terms act as noise within the high-dimensional feature space, causing significant jitter in the loss function surface. They generate deceptive undulations and false valleys, which can misguide the training process and lead the model to capture non-informative correlations. Second, the severe imbalance between positive and negative samples fundamentally distorts the weight distribution of the loss function. This causes the overall loss signal to be dominated by the majority class, while the loss gradient contributed by the scarce yet critical positive samples is suppressed. As a result, the loss surface becomes extremely flat or narrow in critical regions (corresponding to the minority class), while the majority class region may form a dominant, wide, and deep ‘basin’. During descent, the optimizer is naturally drawn toward these dominant basins, thereby neglecting the minority of class regions critical to overall performance. This leads to model failure on key objectives. Furthermore, severe occlusion of the target renders the feature information received by the model incomplete and highly blurred. In feature space, the same object may exhibit distinctly different, discrete distributions depending on the degree and manner of occlusion. This is equivalent to artificially creating multiple isolated yet similar local ‘peaks’ or ‘fragmented troughs’ around the same global optimum on the loss surface. When attempting to approximate the optimal solution, the gradient descent algorithm must jump between these discrete, discontinuous regions. The gradients generated under these conditions are highly unstable and noisy, making it extremely prone to guiding parameter optimization toward a seemingly reasonable yet actually biassed local optimum with poor generalization capabilities.

First, the necessity of the optimization process was analyzed. The WOA’s workflow was then examined, including the improvement plan and the fundamentals of the algorithm’s operation. Then, the working mechanism of the improved WOA proposed in this work was elaborated. Finally, the specific steps for CSAWOA are provided.

**Figure 8:**
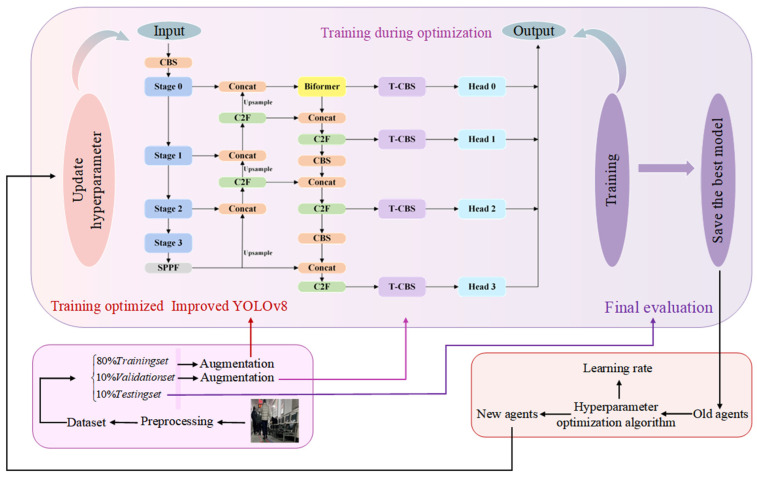
Flowchart for object detection in hospital corridors in complex environments.

The WOA proposed in this work was thoroughly investigated from two perspectives. The first hybrid approach was based on the integration of vertical and cross-search strategies. Second, an improved coefficient updating strategy was introduced to enhance the algorithm’s convergence behaviour and search efficiency. This section details the specific improvements. The CSAWOA optimization process for YOLOv8 is illustrated in Figure 8.

#### 3.2.1. CSAWOA Optimization to Improve the Hyperparameters of the YOLOv8 Model

**Figure 9:**
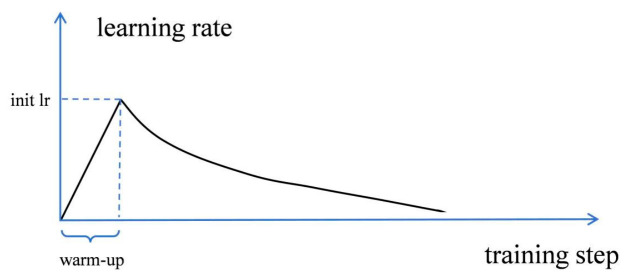
Warm-up strategy diagram.

In many training models, it is necessary to use a warm-up strategy to control the learning rate. At the beginning of network training, the learning rate was slowly increased from a very small value to a set initial learning rate. Then, it was slowly reduced following the normal training process, with an upfront growth from a very small learning rate to an initial value during a phase called warm-up. The authors in ResNet [23] set the initial value to 0.1. During training, a warm-up phase was introduced with an initial learning rate of 0.01. After the loss dropped below 80% of its initial value, the learning rate was adjusted to the initial 0.1 for model optimization, as shown in Figure 9.

Mu [50] obtained all the parameters randomly at the very beginning of the training, which were very far from the results obtained in the final training. Using a very large learning rate right off the bat can lead to unstable values. In other words, the use of a very large learning rate can adversely affect training stability and hinder convergence. Among the networks in this work, momentum modules were used, such as the Adam optimizer, and the learning rate was more dependent on historical statistics. In the early stages of network training, due to the relatively small number of iterations, the statistical history values will be less reliable or simply incorrect. Consequently, the statistical estimates gradually stabilize during training and become reliable only after sufficient iterations have been performed. The learning rate can be slowly increased from a smaller value in the pre-training period, so as to avoid unstable training due to inaccurate historical statistical values at the beginning. Goyalp [51] used an incremental warm-up strategy, where the learning rate grows slowly from 0 to a set initial value, following a linear growth process.

At the beginning of the training process, the network weights are usually randomly initialized, which tends to lead to large fluctuations in the gradient. Learning rate warm-up helps the model find a smoother direction first and avoid large jumps. It is undeniable that the number of warm-up steps needs to be carefully adjusted, and improper selection may affect the model performance. If the warm-up time is too short, the model may not yet reach a smooth state, leading to instability in the early stages of training. Too long warm-up time, in turn, may lead to slower convergence, while too long warm-up of the learning rate can, in some cases, lead to model that is overfit to the training data. The essence of warm-up is to reduce the initial learning rate. However, the initial instability of the model is not fully addressed, especially in the case of poor parameter initialization or complex training data distribution. Gradient oscillations or gradient explosions may still occur during the warm-up phase. Therefore, choosing an appropriate learning rate is crucial.

#### 3.2.2. Overview of WOA

WOA [52] analyzed the search strategies and seining mechanisms of humpback whales, which included three key stages: seining for prey, bubble net feeding and searching for prey. The location of each humpback whale in WOA represents a potential solution, and the global best solution is eventually discovered by continuously updating the whale’s position in the solution space. The main steps are as follows.

1. Initialization: The method starts with a collection of randomly generated whale locations and velocities. These solutions are referred to as whale individuals; that is, they represent possible optimal solutions in the solution space. Additionally, the algorithm’s pertinent parameters are configured, such as the maximum number of iterations and the number of whales.
2. Fitness assessment: Each whale’s fitness value is computed, and the solution quality is evaluated based on the optimization problem’s objective function. A higher fitness value indicates a superior solution.
3. Prey Encirclement: The remaining whales in the population surround the position of the best whale (or a randomly selected whale) to update their positions, under the assumption that the current best individual represents the prey, namely the current optimal solution. Through the use of particular mathematical formulae, this approach enables individual whales to gradually get closer to the ideal answer. Through the use of particular mathematical formulae, this approach enables individual whales to progressively approximate the optimal solution. The following formula for updating a position was used:

(18) X(t+1)=X*(t)−A×D

where A is the shrinkage envelopment coefficient, D refers to the distance between the whale and the prey, and X(t + 1) indicates the location of the whale at the instant t + 1, whereas X*(t) stands for the position of the prey at that moment t (the current optimal solution).

The shrinkage envelopment factor A can be calculated as follows:

(19) A=2×a×rand−a

where a is a linearly decreasing weight whose value decreases with the number of iterations. rand is a random number between 0 and 1. The distance D can be calculated as follows:

(20) D=|C×X*(t)−X(t)|

where C is the following coefficient and X(t) is the position of the whale at moment t.

4. Bubble net predation is the practice of using the swirling swim of a whale around its prey to localized search. Individual whales spiral upwards (current optimal solution) to approach their prey gradually. This stage, which has two sub-steps—spiral update and shrink-wrap—is also accomplished using certain mathematical formulae. The position update formula can be expressed as follows:

(21) $$X(t+1)=D*\times e^{bl}\times cos(2\pi l)+X*(t)$$

(22) D*=|X*(t)−X(t)|

where D* is the absolute value of the distance between the whale and its prey, b is a constant, l is a random number between [−1,1], and b and l are spiral shape control parameters.

5. Search for Prey: To improve exploration and prevent becoming caught in local optima, individual whales perform random searches. Based on their position, whales search and feed at random when certain criteria are satisfied (for example, the absolute value of A is larger than or equal to 1). The positional update formula for encircling prey was used for this step.
6. Update global optimum solution: To direct the subsequent search for individual whales, update the global optimal solution based on the fitness data.
7. Iteration: Repeat the aforementioned actions until the stopping criteria are satisfied, such as reaching the maximum number of iterations or identifying a workable solution.

#### 3.2.3. Horizontal Cross Search

The lateral search mechanism generally takes place between two distinct individuals [53]. This not only facilitates different individuals to share information, but it also makes group exploration even better. As a result, the algorithm’s speed of convergence is increased while simultaneously enhancing population diversity and preventing the algorithm from reaching local optimality. In this work, the horizontal cross search is named ‘C’. Using the individuals X and J of the parent, a lateral search operation in the D-dimension can provide new candidate solution, which can be calculated using Equation (23).

(23) $$\left\{\begin{array}{l} MS_{id}^{hc}=\varepsilon_{1}\times X_{id}+(1-\varepsilon_{1})X_{id}+C_{1}(X_{id}-X_{jd})\\ MS_{jd}^{hc}=\varepsilon_{2}\times X_{jd}+(1-\varepsilon_{2})X_{jd}+C_{2}(X_{jd}-X_{id}) \end{array}\right\}$$

where $MS_{id}^{hc}$ and $MS_{jd}^{hc}$ denote the offspring of the parent after a cross-sectional search in dimension D. $\varepsilon_{1}$ and $\varepsilon_{2}$ are sequential, consistent random integers in the interval (0,1). $C_{1}$ and $C_{2}$ stand for random numbers in the interval (−1,1). To guarantee that the top-performing people are kept after each operation, hence enhancing population quality, they must compete for individuals in the population X of the previous generation after the horizontal crossover operation is finished. The operation is as follows.

(24) $$X=\left\{\begin{array}{l} MS_{hc},ifF(MS_{hc})<F(X)\\ X,ifF(MS_{hc})<F(X) \end{array}\right.$$

#### 3.2.4. Vertical Cross Search

The stagnation of nonconvex OPF problems is improved by vertical cross search [54] and is referred to as ‘S’ in this work. Vertical cross-search functions mainly between different individuals’ many dimensions, in contrast to the horizontal cross-search method previously discussed. In order to continue the search, certain position vectors could become stuck in local optima, while the normal position vectors that are sought stay as constant as possible. Consequently, later in the search phase, whales frequently become stuck at certain location vectors, resulting in a local optimum. By enabling interaction among individuals with diverse search characteristics, this arithmetic crossover procedure prevents premature convergence and enhances the ability of the population to escape local optima. The specific programmes are as follows.

(25) $$MS_{id_{1}}^{vc}=\varepsilon \times X_{id_{1}}+(1-\varepsilon )X_{id_{2}}$$

where ${MS}_{id_{1}}^{vc}$ represents the ith individual in $d_{1}$ dimension ($d_{1}\neq d_{2}$) that is produced by a vertical crossover operation between the parents $X_{id_{1}}$ and $X_{id_{2}}$. ε represents a random integer in the range of 0 to 1.

#### 3.2.5. Adaptive Transformation Strategies

The value of a in conventional WOA may be lowered to zero and linearly declined. This kind of update significantly reduces the algorithm’s global performance and rate of convergence. On the one hand, the value of α is too small, particularly in the latter iterations, which causes the algorithm to be locally optimized. On the other hand, sophisticated high-dimensional optimization goals are not well suited to the linear approximation of α. As a result, the adaptive transformation strategies was added in this work [55]. In this paper known as ‘A’. The improved α is updated as follows.

(26) $$\alpha =-{\left(\frac{2t}{t_{max}}\right)}^{\gamma }+2$$

where the decay parameter is denoted by γ and the number of iterations by t. Additionally, is should be taken into account that in conventional WOA, l is a random variable whose value influences the spiral update’s magnitude. The variation of l was also enhanced to better implement the spiral update approach. l is updated as follows.

(27) $$l=(-c-\frac{t}{t_{max}})\times r+1$$

where r is a random variable in [0,1] and c is a parameter governing the spiral update’s size.

#### 3.2.6. Optimization Process

**Figure 10:**
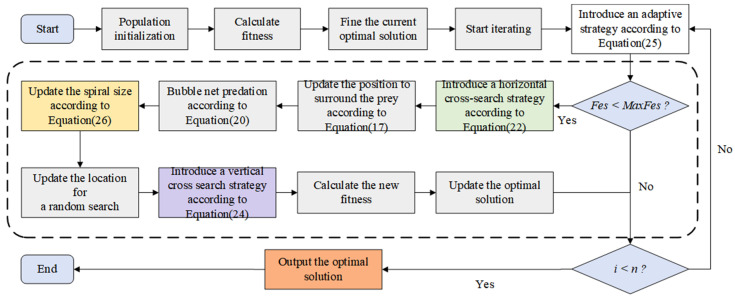
Parameterization of the model using the proposed CSAWOA.

The learning rate of the model was adjusted using the improved CSAWOA, the process of which is shown in Figure 10.

Assuming Z to be the hyperparameter space [0.0001,0.1], the modelling details of the model parameter search are as follows and *n* is the value of the hyperparameter learning rate. Maximizing the diagnosticity of the validation data yields the ideal learning rate, and the objective function for determining the ideal learning rate may be represented as follows.

(28) $$n_{*}=argmax(F),n\in Z$$

where F is the goal function in Equation (27).

The proposed CSAWOA was used to identify the optimal hyperparameter for a single iteration and the hyperparameter was then employed to get the highest value of the real objective function (mAP50 on the validation set). To find the ideal hyperparameters, the model was updated and the previous procedures again was followed. The entire hospital corridor validation procedure is depicted in Figure 10.

Step 1: Data preprocessing. All of the data were downsized to 640 × 640 × 3 and the training data was enlarged using several image enhancement techniques, including flipping in both the horizontal and vertical directions.

Step 2: Using the 8:1:1 ratio, the dataset was randomly divided into three groups: training, validation, and test sets.

Step 3: Train the model. The model was trained using the training data and the learning rate was optimized using CSAWOA. To fully confirm the method’s validity, the experiment was conducted many times.

Step 4: Get the result. The model was fed with test data to allow for the validation of its validity. The ultimate result was obtained by comparing the output with the real results.

## 4. Results

This section first elaborates on the experimental environment. The stability and strong optimization of the CSAWOA algorithm were subsequently demonstrated. Finally, the optimized and improved YOLOv8 model using the CSAWOA algorithm was compared with other classical models. The generalization ability and better detection performance of the method in this work were demonstrated, and the feature map was visualized and output.

### 4.1. Experimental Environment and Parameter Settings

In this work, the training was conducted with a batch size of 32, and the learning rate was adjusted using an improved Whale Optimization Algorithm within the range of [0.001–0.1]. All input images were resized to 640 × 640 × 3. Table 3 provides a summary of the experimental setting. The framework was created using the PyTorch (version 2.1.0) deep learning framework within the Anaconda environment.

### 4.2. CSAWOA Experiment

#### 4.2.1. CSAWOA Strategy Comparison Experiments

To evaluate the effectiveness of the proposed approaches, a comparative study of different strategies and their influence on algorithm performance was conducted. The horizontal cross search strategy is denoted by ‘C’, the vertical cross search strategy is denoted by ‘S’, and the adaptive transformation strategy is denoted by ‘A’, whereas ‘0’ and ‘1’ were used to indicate whether the optimization strategy is adopted or not. More details can be found in Table 4.

The Friedman and Wilcoxon signed rank tests were used in this part. The CSAWOA was tested against the CWOA, SWOA, and AWOA using a single policy, as well as the traditional WOA on 30 functions with the dimensionality of the functions set to 30. By comparing the ‘+/−/=’ superiority and inferiority outcomes produced by various algorithms, as well as the mean optimal value (MEAN) and ranking (RANK), the performance of the algorithms in finding the optimum was examined. Table 5 displays the experimental results.

‘+/−/=’ was used in the table to denote that CSAWOA’s optimization seeking ability is superior to, inferior to, or equal to other algorithms, respectively. CSAWOA outperformed CWOA and SWOA on all 30 functions, outperformed AWOA on 16 functions, and outperformed WOA on 30 functions. Furthermore, MEAN was the optimal value that averages the 30 optimal values that the algorithm produces over 30 separate experiment repeats. The algorithm’s strong optimization ability was indicated by a smaller MEAN, which also indicates that the WOA incorporating the optimization strategy has better optimization ability and search stability. Additionally, the algorithm’s performance can be further improved by combining the two optimization strategies.

#### 4.2.2. CSAWOA Stability Experiments

The function dimensions in this section were 10, 30, 50, and 100, in that order, to compare the outcomes of CSAWOA and WOA in order to examine whether the test function’s dimension change has an impact on the optimization effect of WOA. Table 6 displays the comparison’s findings. Avg represents the CSAWOA and WOA averages, whereas Std represents the standard deviations. The table’s data shows that CSAWOA performed better than WOA in practically every mean and standard deviation function. Furthermore, Table 7 displays the CSAWOA and WOA algorithms’ WSRT and FT test results on the four dimensions. Using the ‘+/−/=’ notation as principle once more, the findings show that CSAWOA performed better than WOA on 29 functions in the 30 dimension and 30 functions in each of the 10, 50, and 100 dimensions. The fact that CSAWOA is ranked number one in every metric provides sufficient evidence suggesting that it performs better than WOA. Overall, CSAWOA is more stable than WOA in every measure, demonstrating its remarkable stability and resilience.

#### 4.2.3. Comparison Experiments Between CSAWOA and Classical Algorithms

To comprehensively evaluate the optimization performance of the proposed CSAWOA, six widely used swarm intelligence optimization algorithms were selected as benchmark methods, including Particle Swarm Optimization (PSO) [56], Ant Colony Optimization (ACO) [57], Moth-Flame Optimization (MFO) [58], Harris Hawks Optimization (HHO) [59], Grey Wolf Optimizer (GWO) [60], and the original Whale Optimization Algorithm (WOA) [52]. In addition, five representative WOA variants, namely improved WOA (IWOA) [61], Adaptive Chaotic WOA (ACWOA) [62], BMWOA [63], CCMWOA [64], and Modified WOA (MWOA) [61], together with the advanced optimization algorithm ALCPSO [65], were included for comparison. These algorithms have been widely applied in continuous optimization and hyperparameter tuning problems and exhibit different exploration–exploitation mechanisms, making them suitable benchmark methods for evaluating the effectiveness and robustness of the proposed CSAWOA. Global optimization experiments on the IEEE CEC2017 test set were also performed, with the experiment configured for 30 dimensions, further validating the performance advantages of the CSAWOA algorithm.

The optimization search results of CSAWOA using other algorithms are shown in Table 8. It is evident from the table’s data that the average CSAWOA value was the lowest of the 20 functions, and the standard deviation value was in the upper level of the 24 methods. This result demonstrates that the CSAWOA algorithm performs well in optimization tests and is the most stable. Using a target function value of 0.05 as the performance benchmark, CSAWOA exhibits a substantial advantage over the competing methods, further confirming its powerful optimization and solution- seeking capability.

The ranks of FT and the ‘+/−/=’ findings of WSRT demonstrated that CSAWOA exhibits varying degrees of superiority when compared to the traditional algorithms. Among them, they were all stronger than WOA, ACWOA, BMWOA, CCMWOA, and MWOA on 30 functions, and all of them showed outstanding advantages on more than half of the functions when compared with PSO, ACO, MFO, HHO, and GWO, in spite of the strong development performance based on the advantages of WOA’s own algorithmic structure. Only ALCPSO offered a bigger challenge to CSAWOA out of the 12 better algorithms; yet, CSAWOA still performed considerably better than ALCPSO in 19 functions, making it the most dominating algorithm on this test set. To sum up, CSAWOA performed exceptionally well in optimization.

**Figure 11:**
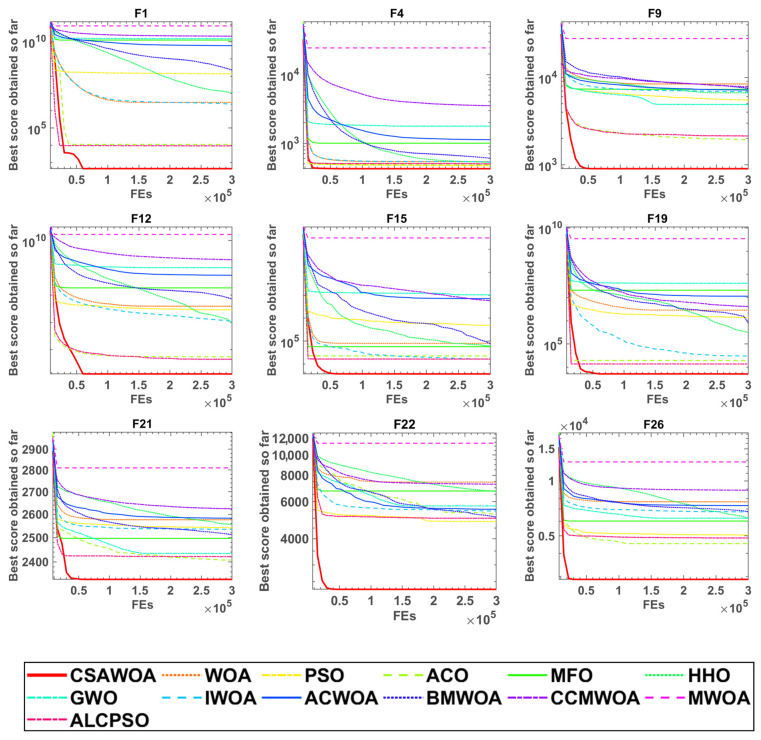
Convergence curves of CSAWOA with different algorithms.

The converged pictures of the 13 algorithms in F1, F4, F9, F12, F15, F19, F21, F22, and F26 are displayed in Figure 11 and Table 9. Visual comparisons of the optimization seeking properties and impacts of various methods can be seen from the figure. The algorithm’s trends in the pre-, mid-, and post-periods were compared and observed using the CSAWOA, which is shown as a bolded red curve. The algorithm’s highest convergence rate is demonstrated by F1 and F12, suggesting that the S-strategy gives the algorithm a significant exploitation advantage. The introduction of the C-strategy endows the algorithm with a better global exploration capacity, as demonstrated by the fact that F22 reaches the convergence phase in the middle and late phases. The red curve is distinguished by both rapid convergence and good convergence accuracy, according to the convergence plots for the other functions. The method’s accuracy was further improved by the addition of the A-strategy, and the F9 and F22 in particular demonstrate that the algorithm performs better in terms of convergence performance than the other algorithms under comparison. Its development capacity was boosted while preserving a robust global exploration capability and avoiding local optimization. All things considered, the CSAWOA optimization algorithm performed better than the other algorithms in this work when it comes to searching for the optimal solution. The convergence curves of the nine benchmark functions collectively demonstrate that the proposed optimization strategies significantly improve the exploration and exploitation capabilities of WOA.

**Table 9:** Optimization results of CSAWOA and other algorithms.

Methods		CSAWOA	WOA	PSO	ACO	MFO	HHO	GWO
WSRT	+/−/=	−/−/−	30/0/0	27/1/2	22/4/4	29/0/1	28/1/1	26/3/1
	Mean	1.53	8.8	5.36	3	7.67	6.6	7.36
	Rank	1	10	4	3	8	6	7
FT	Mean	1.94	8.23	6.01	3.3	7.15	6.72	6.87
	Rank	1	10	4	3	8	6	7
Methods		IWOA	ACWOA	BMWOA	CCMWOA	MWOA	ALCPSO	
WSRT	+/−/=	28/0/2	30/0/0	30/0/0	30/0/0	30/0/0	19/4/7	
	Mean	6.06	9.53	8.03	11.3	13	2.73	
	Rank	5	11	9	12	13	2	
FT	Mean	6.39	9.46	8.10	10.66	12.98	3.13	
	Rank	5	11	9	12	13	2	

### 4.3. CSAWOA-YOLOv8 Experiments

#### 4.3.1. Comparative Experiments with Different Models

To ensure methodological rigour and avoid potential data leakage, the dataset was divided into training, validation, and test sets with a ratio of 8:1:1. During the optimization stage, the proposed CSAWOA algorithm was used only for learning-rate hyperparameter optimization based on the validation-set performance.

More specifically, the training set was used for model parameter learning, while the validation set was employed to evaluate the fitness value of CSAWOA. The fitness/objective function of CSAWOA was defined as the maximization of mAP50 on the validation set rather than the test set. Through iterative optimization, CSAWOA automatically searched for the optimal learning-rate parameter combination that achieved the best validation performance.

After all hyperparameters were determined, the optimized model was finally evaluated on the independent test set. The test data were used only once for the final unbiased performance evaluation, including mAP, precision, and recall.

Therefore, the test set was completely excluded from the hyperparameter optimization process, ensuring strict separation between training, validation, and testing stages and preventing potential evaluation bias caused by test-set leakage.

To confirm the detection capabilities of the CSAWOA-YOLOv8 framework, the same dataset gathered for this work was used. The effectiveness of the CSAWOA approach was initially assessed using five distinct models. The optimal learning rate for model training was explored using the CSAWOA optimization method between [0.0001,0.1], as shown in Table 10. Following five iterations of cross-validation, the framework suggested in this research exhibited a mean mAP50 value of 76.2%. It is evident that employing varying learning rates does yield a broad range of outcomes. The mAP50 results for YOLOv10 differ significantly between learning rates of 0.008 and 0.07. The experimental results demonstrate a clear degradation in performance as the selected learning rate increasingly deviates from the optimal value. Additionally, it was found that the detection results are poorer when the learning rate is approaching 0.1. This is due to the CSAWOA search interval’s upper limit being set at 0.1. According to the earlier study, a learning rate greater than 0.01 adversely affects the detection capability of the model. Since 0.1 was the highest value that CSAWOA reached, learning rates around 0.1 yielded unsatisfactory outcomes.

**Figure 12:**
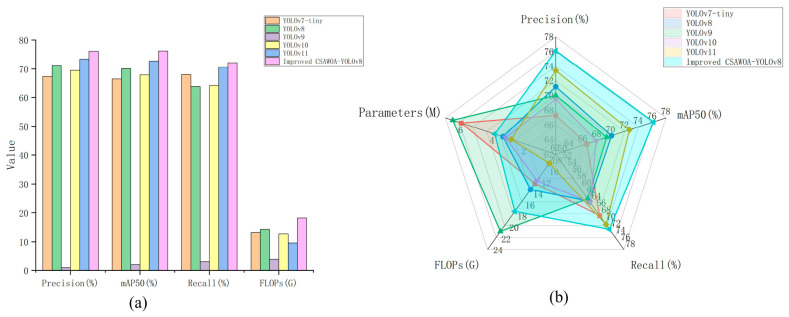
Comprehensive comparison of seven algorithms. (**a**) Histogram format. (**b**) Pentagonal representation.

Table 11 shows a further comparison of CSAWOA-YOLOv8 with the four optimized CSAWOA models and the original YOLOv8 model in other aspects. Compared to the original YOLOv8, the CSAWOA-YOLOv8 model achieved improvements of 4.9%, 6.1%, and 8.3% in accuracy, mAP, and recall, respectively. In terms of parameters and FLOPs, it is undeniable that the proposed framework was indeed larger than the original YOLOv8 model and YOLOv11; however, the model size remained within an acceptable range. At the same time, the framework proposed in this work demonstrated a significant improvement in the detection performance compared to YOLOv11. The experimental setup for this experiment was the same as described above. The FPS metric was also included in our evaluation. As evidenced by the experimental results, the proposed model achieved a higher FPS score compared to the baseline model. Histograms were also deployed for comparison in order to further illustrate the superior detection performance of the proposed model in this work. As shown in Figure 12a, the CSAWOA-YOLOv8 exhibited the best detection performance. Additionally, as can be seen in Figure 12b, radargrams were employed to synthesize their detection performance. In the radar diagram, algorithm performance improves as the enclosed polygon area increases, whereas greater radial distances from the centre indicate stronger performance on individual evaluation metrics. It is evident that the CSAWOA-YOLOv8 framework introduced in this work has better overall metrics than the previous models. The improvements result in a larger model with additional parameters; however, the resulting gains in deployment performance justify the increased computational cost.

When the proportion of samples in the minority class was extremely low, the majority class was sufficient to skew the overall mean upwards, resulting in an artificially inflated improvement in the overall metrics. To verify whether the improved Focal Loss, T-CBS and BiFormer modules proposed in this work truly resolve the issue of class imbalance, a systematic analysis from two perspectives was conducted in this section: per-class detection metrics and the normalized confusion matrix.

Table 12 presents a comparison of the P, R and mAP50 values for the original YOLOv8 and our proposed CSAWOA-YOLOv8 across various categories. As can be observed, for most classes, the mAP50 for ‘Person’ was improved from 83.5% to 85.5%, for ‘Chair’ from 78.5% to 81.5%, and for ‘Handbag’ from 79.0% to 82.5%. As these classes already exhibit high detection accuracy in the baseline model, the scope for improvement was relatively limited. However, for a few classes, the increase in mAP50 was significantly amplified: Trash bin improved by 11.0% and Fire hydrant by 12.0%, with the recall rates for these two classes rising from 53.0% to 65.0% and from 48.0% to 62.0%, respectively. It is worth noting that the mAP50 improvement for ‘sVehicle’ was +5.1%, falling between that of the majority classes and the other two minority classes. This class encompasses a variety of heterogeneous targets, such as trolleys, wheelchairs and medical trolleys. Although the sample size is comparable to that of ‘Trash bin’ and Fire hydrant, the significant morphological variation within the class prevents the category-weighted mechanism of the improved Focal Loss from functioning as effectively as it does for minority classes with relatively uniform morphology; nevertheless, the gain remains markedly higher than that of the majority classes, indicating that the method proposed in this work is equally effective for minority classes with high intra-class variance. Overall, the mAP50 improvement for minority classes reached +9.4%, far exceeding the +2.8% for majority classes, indicating that the overall +6.1% increase in mAP was not driven by majority classes; rather, minority classes were the primary beneficiaries of the improvements.

**Figure 13:**
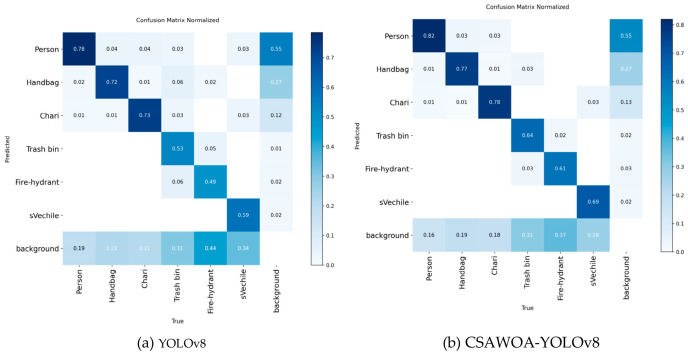
Confusion matrix.

Figure 13 shows the normalized confusion matrices for the two models on the test set. In the original YOLOv8, the false negative rates for minority classes were significantly high, reaching 44% for ‘Fire-hydrant’, 34% for ‘sVehicle’ and 31% for ‘Trash bin’, reflecting the baseline model’s strong suppression of minority classes under class-imbalanced conditions. Furthermore, due to the morphological similarity between Fire hydrant and Trash bin, significant cross-class confusion between the two was detected: 6% of Trash bins were misclassified as Fire hydrants, and 5% of Fire hydrants were misclassified as Trash bins. In CSAWOA-YOLOv8, the proportions of Fire hydrant and sVehicle incorrectly classified as background decreased to 37% and 28%, respectively, whilst cross-class confusion simultaneously fell to 3% and 2%. The diagonal values for the major classes were also improved: for people, from 0.78 to 0.82; for seats, from 0.73 to 0.78; and for handbags, from 0.72 to 0.77. This result indicates that the improved Focal Loss, through adaptive adjustment of class weights, has focused on enhancing the diagonal values (i.e., recall) for minority classes, thereby alleviating the suppression of minority classes by the background. Meanwhile, the BiFormer’s sparse attention mechanism enables the model to distinguish morphologically similar objects based on contextual information, reducing cross-class confusion. The modest improvement for major classes primarily stems from the global context modelling introduced by T-CBS, which alleviates the occlusion issues commonly encountered in corridor scenarios.

#### 4.3.2. Ablation Experiments

This experiment compares the object detection performance of the original YOLOv8 model and three improved versions on the dataset. The improved versions incorporate the T-CBS module, the BiFormer module and the CSAWOA algorithm, respectively. The main evaluations included precision (P), recall (R), mAP50 and mAP50-90. A comparison of the original model and improved framework is shown in Table 13.

**Figure 14:**
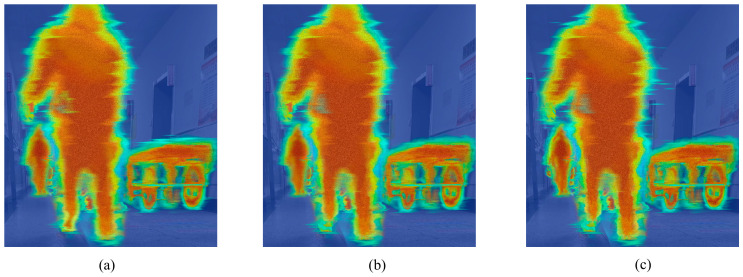
Comparison of feature maps from different modules. (**a**) Feature map from the YOLOv8 backbone network. (**b**) Feature map after adding T-CBS alone. (**c**) Feature map after adding the BiFormer module alone.

The experimental results show that, compared to the original YOLOv8 model, the addition of the T-CBS module improved P, R, mAP50, and mAP50-90 by 1.8%, 1.4%, 1.5%, and 3.5%, respectively. As can be seen, the T-CBS proposed in this work effectively addressed the problem of object occlusion. When an object is occluded, certain parts of it are not visible. However, the attention mechanism allows the visible part of the target to directly focus on other parts of the same target, or even on contextual information in the scene that typically aligns with the target. The T-CBS architecture proposed in this work incorporates a self-attention mechanism and, through global inference, enables each object query to identify a complete and unique target within the global context, rather than relying solely on local features. This helps the model distinguish between objects that are closely adjacent in pixels but belong to different instances. However, given the wide variety of recognition tasks in hospital corridors—with numerous small objects of varying sizes—simply applying the aforementioned improvements to the original model is insufficient to fully meet practical needs, leaving considerable room for improvement in detection performance. Therefore, the BiFormer module was incorporated. It divides the input image into multiple regions and, through region-to-region routing, quickly filters out a small number of candidate regions that may be relevant to each query region, directly eliminating a large number of obviously irrelevant background regions. Subsequently, more refined token-to-token attention calculations were performed. For small objects, this mechanism ensures that their limited visible features can efficiently interact with the most relevant contextual information (such as other visible parts of the same object) without being distracted by large objects or complex corridor environments, helping small objects leverage richer detection details. After incorporating the improved Focal Loss function, the mAP50 value increased by 1.6% compared to the original YOLOv8 model. After incorporating the CSAWOA strategy, the final improved framework achieved an mAP50 score of 76.6. Feature maps at various phases were also independently produced, as illustrated in Figure 14, to further confirm the suggested T-CBS and BiFormer modules’ efficacy in improving feature representations from a mechanical standpoint. It is clear that the T-CBS module outperforms the original YOLOv8 in detecting the outlines of occluded objects. Nonetheless, the BiFormer module concentrates on the structural areas of tiny targets and shows a decrease in the activation responses in background regions.

#### 4.3.3. Comparative Experiment of Improved Loss Functions

**Figure 15:**
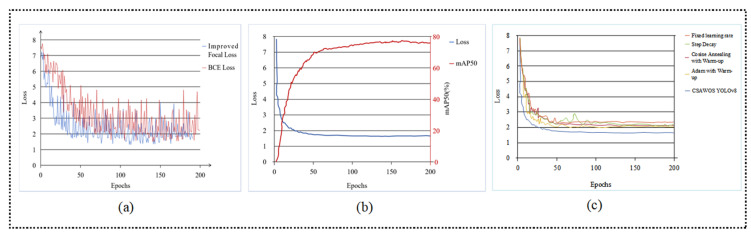
(**a**) Comparison of two loss functions. (**b**) CSAWOA-YOLOv8 framework training results diagram. (**c**) Comparison of scheduling strategies for different learning rates.

The improved Focal Loss function and the BCE function from the original work were compared to train the model. Figure 15a shows that the training process is more stable, the declining trend is smoother, and the enhanced loss function hits the minimum point sooner and the loss value falls. The proposed function offers stronger generalization and robustness compared to the original function, thereby contributing to better detection performance.

Figure 15b shows the progression of the loss function value and mAP50 during framework training. The framework exhibited a high learning rate at the start of training since the CSPWOA method was used, and the loss function value rapidly decreased over the first 50 epochs. The loss function values gradually decreased as training went on, stabilizing at epoch 200 and varying at 1.78. The mAP50 number, on the other hand, quickly increased throughout the first 50 epochs before progressively levelling off and ultimately varying about 76.8%.

Figure 15c compares the scheduling strategies with different learning rates. As can be ascertained, the proposed CSAWOA achieved faster convergence, transitioned more smoothly to the high-learning-rate region compared to a fixed learning rate, and avoided initial oscillations. Lower final loss enabled CSAWOA to explore the loss surface more precisely during the later stages of training, identifying optimal minima points and avoiding stagnation in plateaus caused by fixed learning rates. Better stability: Although the other methods are prone to oscillation during late training stages due to excessively high learning rates, the CSAWOA mechanism smoothly reduces the learning rate to ensure stable convergence.

#### 4.3.4. Feature Map Visualization Analysis

**Figure 16:**
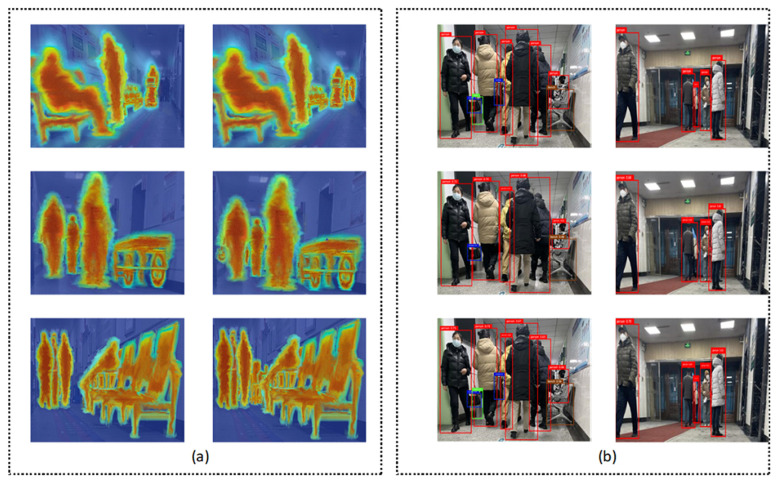
Visual analytics. (**a**) The left column of the figure is the thermal map of the original YOLOv8 and the right column is the thermal map of the CSAWOA-YOLOv8. (**b**) The first row shows the real label of the image, the second row shows the YOLOv8 detection result, and the last row shows the CSAWOA-YOLOv8 detection result.

The tiny target output layers of YOLOv8 and CSAWOA-YOLOv8 were shown using gradient-weighted class activation mapping to show the comparison between before and after model optimization [66]. Without improving YOLOv8, the model suffers from overlapping targets and other complicated backdrops, as can be seen in Figure 16a; it is unable to properly capture all objects and has trouble focusing on smaller targets. However, the network concentrate more on regions of detail with CSAWOA-YOLOv8, making the target’s edge information more noticeable. This effect implies that feature augmentation enables the network to completely exploit contextual information in order to record pedestrians’ states and objects in hospital hallways. The model’s detection performance may be enhanced by optimally modifying the learning rate, which allows the model to modify the feature weights and extract the target’s essential features more precisely while suppressing other unnecessary background interference. On the feature map, each pixel value corresponds to the activation value at that particular position. As the activation value increases, the model becomes more confident that the corresponding region contains the target object. As a result, the region appears brighter and more prominent in the heat map.

Figure 16b shows the detection results of the framework on the test image. The image’s true label appears on the first line, followed by the YOLOv8 detection result and the framework’s detection result, which is the last line in this work. The initial YOLOv8 model exhibited poor performance results and underdetection, as can be seen in Figure 16b. The improved model successfully increased the detection accuracy by having a greater detection accuracy for human and object detection of tiny targets and overlapping regions in complicated backdrops and the same scene.

## 5. Discussion

This study utilized the 2023 ICV Algorithm Challenge dataset to validate the generalization capability of the framework [67]; this dataset comprises images of urban road scenes captured by monocular cameras mounted on vehicles. In this work, the proposed framework, along with other model frameworks, was retrained on this dataset, with training repeated using multiple random seeds. The final experimental results are shown in Table 14. The table indicates that the framework proposed in this work achieved a higher mAP value than the other models and a significantly higher recall rate, suggesting that the model is not only capable of detecting a large number of small objects but also of accurately identifying many positive samples. This result demonstrates that the framework adopted in this work possesses excellent detection and generalization capabilities.

Although the proposed CSAWOA-YOLOv8 framework achieved improved detection performance and robustness in complex hospital corridor environments, several limitations still exist. First, the current framework fundamentally belongs to a fully supervised learning paradigm and therefore relies heavily on manually annotated bounding-box datasets. In real hospital scenarios, collecting and annotating large-scale high-quality datasets is both time-consuming and labour-intensive. Furthermore, the proposed T-CBS, BiFormer, and CSAWOA modules, while improving feature extraction capability and optimization stability, may also increase the model’s dependence on sufficient annotated data for effective training.

To alleviate this issue, several strategies were deployed, including data augmentation, transfer learning, adaptive category-weight adjustment, and the improved Focal Loss mechanism, to improve the utilization efficiency of limited labelled samples. The experimental results demonstrated that these strategies effectively enhanced minority-class detection and improved robustness under occlusion and complex environmental conditions. However, these approaches still operate within the scope of supervised learning and do not fundamentally eliminate the requirement for annotated data.

Another important limitation is the potential influence of domain shift. Different hospital environments may exhibit substantial variations in camera parameters, corridor layouts, lighting conditions, obstacle distributions, and pedestrian behaviours. As a result, models trained on a specific hospital dataset may experience performance degradation when deployed in unseen environments. Although the generalization experiments on the 2023 China ICV Algorithms Challenge dataset verified that the proposed framework retains relatively good adaptability after fine-tuning, the current study still requires supervised retraining or parameter adaptation on the target dataset.

Therefore, future work should focus on developing more label-efficient and cross-domain generalizable learning frameworks. In particular, domain adaptation-based approaches may provide a promising direction for improving practical deployment capability while reducing annotation requirements [68]. Potential future research directions include unsupervised learning, semi-supervised domain adaptation for object detection [69], self-supervised pretraining, active learning, and few-shot detection methods. Combining these approaches with the proposed T-CBS feature extraction module and adaptive category-weight optimization strategy may further enhance model generalization and robustness across different hospital environments while reducing dependence on large-scale labelled datasets.

Although CSAWOA was employed as a hyperparameter optimization algorithm for YOLOv8, its significance extends beyond a conventional optimization tool. CSAWOA was derived from the Whale Optimization Algorithm (WOA), which mimics humpback whales’ hunting behaviours, including prey encircling, spiral bubble-net attacking, and prey searching. These biologically inspired mechanisms provide an effective balance between global exploration and local exploitation.

To overcome the limitations of the original WOA, such as premature convergence and insufficient population diversity, CSAWOA introduces horizontal cross search, vertical cross search, and an adaptive transformation strategy. From a biomimetic perspective, these mechanisms enhance information exchange, cooperative search, and adaptive behavioural adjustment within the population, thereby improving optimization efficiency and robustness.

Therefore, CSAWOA is not merely a metaheuristic optimizer applied to YOLOv8, but an extension of whale-inspired collective intelligence. By enhancing population interaction and adaptive search behaviours, CSAWOA achieved better exploration–exploitation balance, faster convergence, and improved optimization performance in complex engineering applications. This study therefore contributes not only to development of intelligent optimization techniques for object detection, but also to the broader field of biologically inspired computational strategies, providing a robust framework for addressing complex optimization problems in real-world environments.

## 6. Conclusions

Although AGVs have been widely developed and applied, achieving fully autonomous navigation in hospital corridors remains a critical and challenging task. To effectively address the issues of complex corridor environments and the difficulty of detecting small targets in such environments, a CSAWOA-YOLOv8 framework was introduced in this work. By introducing the T-CBS module and BiFormer module, the global background and local features were effectively extracted, and the focus on background information was reduced to improve the detection performance of small targets. Meanwhile, the improved Focal Loss function solved the class imbalance problem in the complex corridor environments and improved the performance of the model. In addition, the proposed CSAWOA algorithm further enhanced the model’s capacity for detection and generalization by optimizing the learning rate of the model. The outcomes of the experiment demonstrate that in comparison with the other five SOTA models, the framework proposed in this work exhibited higher mAP, precision and recall, which were: 76.1%, 76.2% and 72.1%, respectively. These results highlight the effectiveness of the proposed approach. However, the methodology still faces limitations, such as the need to reduce the number of experimental parameters. In future work, this framework will be combined with binocular ranging technology to enable AGVs to achieve reliable obstacle avoidance. The development of unmanned autonomous driving technology for AGVs represents a unique opportunity to streamline and transform hospital operations. The integration of such mobile robotic solutions is expected to enhance patient care, improve operational efficiency, and reshape modern healthcare environments. Ultimately, the proposed algorithm is envisioned to be extended to a broader range of intelligent transportation systems to further advance autonomous driving technology.

## Figures and Tables

**Table 3:** Experimental environment configuration.

Environment Configuration	Parameter
Operating System	Linux
CPU	Ntel (R) Xeon (R) Gold 6148 CPU @2.40 GHz
GPU	2 × A100 (80 GB)
Development environment	PyCharm 2023.2.5
Language	Python 3.8.10
Framework	PyTorch 2.0.1
Operating platform	CUDA 11.8

**Table 4:** Expressions of MFO with different strategies.

	C	S	A
WOA	0	0	0
CWOA	1	0	0
SWOA	0	1	0
AWOA	0	0	1
CSAWOA	1	1	1

**Table 5:** Comparative results of WOA in WSRT and FT with different strategies.

	+/−/=	MEAN	RANK	ARV	RANK
CSAWOA	~	1.33	1	1.36	1
CWOA	30/0/0	3.20	3	3.46	3
SWOA	30/0/0	3.96	4	3.93	4
AWOA	16/7/7	1.73	2	1.75	2
WOA	30/0/0	4.76	5	4.48	5

**Table 6:** Mean value and standard deviation in different dimensions.

Fun		dim = 10		dim = 30	
		CSAWOA	WOA	CSAWOA	WOA
F1	Avg	3.246706 × 10^4^	6.416658 × 10^7^	4.707382 × 10^2^	2.923246 × 10^6^
	Std	1.647538 × 10^4^	3.471910 × 10^7^	5.953029 × 10^2^	2.001501 × 10^6^
F2	Avg	1.852676 × 10^21^	1.829972 × 10^30^	2.305007 × 10^8^	6.565685 × 10^20^
	Std	4.467697 × 10^21^	1.002209 × 10^31^	1.009311 × 10^9^	2.488035 × 10^21^
F3	Avg	6.986769 × 10^4^	2.167632 × 10^5^	2.256570 × 10^3^	1.576490 × 10^5^
	Std	5.966553 × 10^3^	7.810693 × 10^4^	1.394265 × 10^3^	5.588610 × 10^4^
F4	Avg	4.410959 × 10^2^	6.039547 × 10^2^	4.248915 × 10^2^	5.358490 × 10^2^
	Std	2.609098 × 10^1^	6.157835 × 10^1^	1.419578 × 10^1^	2.925275 × 10^1^
F5	Avg	7.137121 × 10^2^	7.938224 × 10^2^	6.804969 × 10^2^	7.745402 × 10^2^
	Std	2.496310 × 10^1^	6.691056 × 10^1^	1.852377 × 10^1^	5.593161 × 10^1^
F6	Avg	6.046968 × 10^2^	6.729814 × 10^2^	6.000141 × 10^2^	6.689669 × 10^2^
	Std	1.505432	1.072843 × 10^1^	4.049758 × 10^−3^	1.300955 × 10^1^
F7	Avg	9.581805 × 10^2^	1.257397 × 10^3^	9.200118 × 10^2^	1.240644 × 10^3^
	Std	2.254536 × 10^1^	7.769062 × 10^1^	1.963948 × 10^1^	8.725026 × 10^1^
F8	Avg	9.936177 × 10^2^	1.016192 × 10^3^	9.839243 × 10^2^	1.000034 × 10^3^
	Std	8.227575	4.279894 × 10^1^	1.817861 × 10^1^	4.208195 × 10^1^
F9	Avg	1.491948 × 10^3^	9.663874 × 10^3^	9.014014 × 10^2^	7.464890 × 10^3^
	Std	3.778568 × 10^2^	3.780623 × 10^3^	1.263472	2.231017 × 10^3^
F10	Avg	5.375994 × 10^3^	6.680201 × 10^3^	5.389522 × 10^3^	6.410936 × 10^3^
	Std	2.086287 × 10^2^	9.303383 × 10^2^	2.045432 × 10^2^	8.980787 × 10^2^
F11	Avg	1.700348 × 10^3^	2.745461 × 10^3^	1.198498 × 10^3^	1.475480 × 10^3^
	Std	1.738224 × 10^2^	1.229117 × 10^3^	1.812738 × 10^1^	1.573119 × 10^2^
F12	Avg	6.598710 × 10^6^	6.781059 × 10^7^	9.598601 × 10^4^	4.085498 × 10^7^
	Std	5.115002 × 10^6^	4.925315 × 10^7^	6.923239 × 10^4^	2.741172 × 10^7^
F13	Avg	6.027742 × 10^4^	2.160045 × 10^5^	9.826008 × 10^3^	1.818836 × 10^5^
	Std	9.106248 × 10^4^	1.196838 × 10^5^	6.562421 × 10^3^	8.790256 × 10^4^
F14	Avg	1.361439 × 10^5^	1.848696 × 10^6^	2.870253 × 10^3^	8.805638 × 10^5^
	Std	1.129324 × 10^5^	2.144050 × 10^6^	1.621053 × 10^3^	9.230000 × 10^5^
F15	Avg	1.715943 × 10^4^	1.183360 × 10^5^	2.784162 × 10^3^	7.617225 × 10^4^
	Std	2.228228 × 10^4^	1.425281 × 10^5^	1.363528 × 10^3^	5.334408 × 10^4^
F16	Avg	2.731932 × 10^3^	3.848341 × 10^3^	1.948544 × 10^3^	3.564106 × 10^3^
	Std	3.559869 × 10^2^	5.037712 × 10^2^	2.161750 × 10^2^	4.453139 × 10^2^
F17	Avg	2.021920 × 10^3^	2.610941 × 10^3^	1.895960 × 10^3^	2.554314 × 10^3^
	Std	1.052611 × 10^2^	2.602740 × 10^2^	8.452329 × 10^1^	2.411554 × 10^2^
F18	Avg	6.331131 × 10^5^	5.238916 × 10^6^	4.293264 × 10^4^	2.696380 × 10^6^
	Std	4.382794 × 10^5^	5.056911 × 10^6^	1.617586 × 10^4^	3.212312 × 10^6^
F19	Avg	2.209150 × 10^4^	5.062733 × 10^6^	5.298405 × 10^3^	3.032661 × 10^6^
	Std	2.125668 × 10^4^	3.861326 × 10^6^	3.178810 × 10^3^	2.824725 × 10^6^
F20	Avg	2.525966 × 10^3^	2.834404 × 10^3^	2.343360 × 10^3^	2.693518 × 10^3^
	Std	1.329572 × 10^2^	1.733348 × 10^2^	7.237017 × 10^1^	2.107914 × 10^2^
F21	Avg	2.407655 × 10^3^	2.604741 × 10^3^	2.329457 × 10^3^	2.572095 × 10^3^
	Std	5.344180 × 10^1^	6.151696 × 10^1^	6.355167	5.260310 × 10^1^
F22	Avg	2.388347 × 10^3^	7.451328 × 10^3^	2.300374 × 10^3^	7.182519 × 10^3^
	Std	1.585964 × 10^2^	1.959003 × 10^3^	1.380688	1.773601 × 10^3^
F23	Avg	2.832856 × 10^3^	3.057639 × 10^3^	2.741815 × 10^3^	6.280784 × 10^1^
	Std	4.547461 × 10^1^	9.996584 × 10^1^	3.035825 × 10^3^	7.829431 × 10^1^
F24	Avg	2.957470 × 10^3^	3.171146 × 10^3^	2.850379 × 10^3^	3.180005 × 10^3^
	Std	7.173205 × 10^1^	9.425579 × 10^1^	6.984617	8.339588 × 10^1^
F25	Avg	2.895227 × 10^3^	3.006760 × 10^3^	2.878381 × 10^3^	2.949466 × 10^3^
	Std	1.664464 × 10^1^	4.062648 × 10^1^	6.446613 × 10^−1^	2.893333 × 10^3^
F26	Avg	2.900244 × 10^3^	7.940917 × 10^3^	2.893333 × 10^3^	7.468780 × 10^3^
	Std	2.597624 × 10^1^	8.764491 × 10^2^	2.537081 × 10^1^	1.102820 × 10^3^
F27	Avg	3.209396 × 10^3^	3.410826 × 10^3^	3.201534 × 10^3^	3.342999 × 10^3^
	Std	1.768619 × 10^1^	1.417167 × 10^2^	4.720399	8.925397 × 10^1^
F28	Avg	3.260870 × 10^3^	3.386864 × 10^3^	3.196170 × 10^3^	3.303122 × 10^3^
	Std	1.844004 × 10^1^	6.120108 × 10^1^	4.347228 × 10^1^	2.525694 × 10^1^
F29	Avg	3.805085 × 10^3^	4.955562 × 10^3^	3.558016 × 10^3^	4.858650 × 10^3^
	Std	1.784035 × 10^2^	4.747935 × 10^2^	1.220473 × 10^2^	4.004479 × 10^2^
F30	Avg	6.098738 × 10^5^	1.929366 × 10^7^	2.707518 × 10^4^	1.214677 × 10^7^
	Std	7.498299 × 10^5^	2.142547 × 10^7^	3.959956 × 10^4^	7.633034 × 10^6^
Fun		dim = 50		dim = 100	
		CSAWOA	WOA	CSAWOA	WOA
F1	Avg	1.000000 × 10^2^	6.221319 × 10^5^	1.000000 × 10^2^	7.160251 × 10^4^
	Std	2.043394 × 10^−13^	6.383085 × 10^5^	6.981845 × 10^−15^	9.005781 × 10^4^
F2	Avg	2.137879 × 10^2^	2.699248 × 10^18^	2.000001 × 10^2^	6.012855 × 10^16^
	Std	2.478538 × 10^1^	4.564857 × 10^18^	5.597713 × 10^−5^	1.508434 × 10^17^
F3	Avg	3.000000 × 10^2^	1.204268 × 10^5^	3.000000 × 10^2^	8.117508 × 10^4^
	Std	4.382194 × 10^−8^	5.823776 × 10^4^	2.360294 × 10^−14^	4.494471 × 10^4^
F4	Avg	4.149361 × 10^2^	5.312237 × 10^2^	4.011383 × 10^2^	5.163473 × 10^2^
	Std	1.533987	3.417419 × 10^1^	1.724742	2.781198 × 10^1^
F5	Avg	6.598770 × 10^2^	7.995182 × 10^2^	6.405495 × 10^2^	7.799536 × 10^2^

**Table 7:** Ranking of WSRT and FT in different dimensions.

		dim = 10		dim = 30		dim = 50		dim = 100	
		CSAWOA	WOA	CSAWOA	WOA	CSAWOA	WOA	CSAWOA	WOA
WSRT	+/−/=	−/−/−	30/0/0	−/−/−	29/0/1	−/−/−	30/0/0	−/−/−	30/0/0
	Mean	1	2	1	2	1	2	1	2
	Rank	1	2	1	2	1	2	1	2
FT	Mean	1.04	1.95	1.02	1.97	1.02	1.97	1.01	1.98
	Rank	1	2	1	2	1	2	1	2

**Table 8:** Optimization results of CSAWOA and other algorithms in 30-dimension.

	F1		F2		F3	
	Avg	Std	Avg	Std	Avg	Std
CSAWOA	4.394377 × 10^2^	4.776026 × 10^2^	7.549503 × 10^7^	3.164841 × 10^8^	2.681104 × 10^3^	1.674237 × 10^3^
WOA	3.006888 × 10^6^	1.943802 × 10^6^	8.890748 × 10^20^	2.423664 × 10^21^	1.741167 × 10^5^	6.897220 × 10^4^
PSO	1.341291 × 10^8^	1.318661 × 10^7^	4.887724 × 10^13^	5.058293 × 10^13^	6.278181 × 10^2^	4.137372 × 10^1^
ACO	1.109662 × 10^4^	7.901486 × 10^3^	1.006066 × 10^33^	5.510451 × 10^33^	1.126666 × 10^4^	1.501892 × 10^4^
MFO	1.210898 × 10^10^	8.540475 × 10^9^	1.223314 × 10^39^	6.539406 × 10^39^	8.643269 × 10^4^	5.055742 × 10^4^
HHO	1.061109 × 10^7^	1.967052 × 10^6^	2.656422 × 10^12^	5.436622 × 10^12^	4.713763 × 10^3^	1.765733 × 10^3^
GWO	1.454278 × 10^10^	6.126589 × 10^9^	2.792080 × 10^37^	1.522909 × 10^38^	4.904915 × 10^4^	9.657804 × 10^3^
IWOA	2.270930 × 10^6^	2.165246 × 10^6^	1.979457 × 10^18^	7.117310 × 10^18^	8.292604 × 10^4^	3.249330 × 10^4^
ACWOA	5.808351 × 10^9^	3.085468 × 10^9^	1.310910 × 10^34^	5.026668 × 10^34^	4.971960 × 10^4^	1.010069 × 10^4^
BMWOA	2.228745 × 10^8^	9.412860 × 10^7^	5.663676 × 10^24^	2.951566 × 10^25^	7.129994 × 10^4^	8.521236 × 10^3^
CCMWOA	2.047150 × 10^10^	5.739729 × 10^9^	3.366310 × 10^40^	1.823317 × 10^41^	7.813093 × 10^4^	3.775373 × 10^3^
MWOA	7.972852 × 10^10^	1.984969 × 10^10^	1.507767 × 10^48^	6.548795 × 10^48^	1.620461 × 10^8^	5.551596 × 10^8^
ALCPSO	9.179903 × 10^3^	7.511339 × 10^3^	3.658597 × 10^18^	1.822389 × 10^19^	2.569211 × 10^4^	4.354086 × 10^3^
	F4		F5		F6	
	Avg	Std	Avg	Std	Avg	Std
CSAWOA	4.265527 × 10^2^	1.761222 × 10^1^	6.870234 × 10^2^	1.267344 × 10^1^	6.000175 × 10^2^	9.372069 × 10^−3^
WOA	5.384121 × 10^2^	3.096795 × 10^1^	7.831403 × 10^2^	6.137129 × 10^1^	6.725115 × 10^2^	1.387714 × 10^1^
PSO	4.704643 × 10^2^	3.695373 × 10^1^	7.497177 × 10^2^	2.376426 × 10^1^	6.558305 × 10^2^	1.362787 × 10^1^
ACO	4.850582 × 10^2^	1.843175 × 10^1^	6.194611 × 10^2^	6.168373 × 10^1^	6.026131 × 10^2^	3.259537
MFO	1.002016 × 10^3^	5.380578 × 10^2^	7.108371 × 10^2^	3.906711 × 10^1^	6.386681 × 10^2^	1.296286 × 10^1^
HHO	5.281051 × 10^2^	3.526685 × 10^1^	7.296763 × 10^2^	3.293071 × 10^1^	6.624339 × 10^2^	5.745084
GWO	1.773182 × 10^3^	1.412090 × 10^3^	6.568785 × 10^2^	2.893311 × 10^1^	6.291648 × 10^2^	1.032564 × 10^1^
IWOA	5.352491 × 10^2^	3.575322 × 10^1^	7.544158 × 10^2^	5.106384 × 10^1^	6.525665 × 10^2^	8.116926
ACWOA	1.128490 × 10^3^	5.449657 × 10^2^	8.030384 × 10^2^	2.960112 × 10^1^	6.665605 × 10^2^	6.835687
BMWOA	6.068198 × 10^2^	5.034653 × 10^1^	7.796323 × 10^2^	4.642584 × 10^1^	6.654500 × 10^2^	9.013591
CCMWOA	3.524171 × 10^3^	1.415084 × 10^3^	8.284976 × 10^2^	3.081124 × 10^1^	6.699613 × 10^2^	7.844472
MWOA	2.442413 × 10^4^	9.724215 × 10^3^	1.034249 × 10^3^	6.022103 × 10^1^	7.177009 × 10^2^	1.443363 × 10^1^
ALCPSO	5.023341 × 10^2^	2.957724 × 10^1^	5.978616 × 10^2^	2.556669 × 10^1^	6.057152 × 10^2^	6.277318
	F7		F8		F9	
	Avg	Std	Avg	Std	Avg	Std
CSAWOA	9.205945 × 10^2^	1.843070 × 10^1^	9.824075 × 10^2^	1.699440 × 10^1^	9.013970 × 10^2^	1.616767
WOA	1.251352 × 10^3^	8.714439 × 10^1^	1.016389 × 10^3^	5.886092 × 10^1^	8.410921 × 10^3^	2.744220 × 10^3^
PSO	9.249774 × 10^2^	1.833086 × 10^1^	9.857202 × 10^2^	2.361120 × 10^1^	5.511073 × 10^3^	1.897623 × 10^3^
ACO	9.394099 × 10^2^	1.014254 × 10^2^	9.200513 × 10^2^	6.236757 × 10^1^	1.932963 × 10^3^	1.291250 × 10^3^
MFO	1.129782 × 10^3^	1.841640 × 10^2^	1.021831 × 10^3^	5.219445 × 10^1^	7.298917 × 10^3^	2.219624 × 10^3^
HHO	1.241842 × 10^3^	7.852826 × 10^1^	9.673396 × 10^2^	2.359177 × 10^1^	6.669863 × 10^3^	7.812051 × 10^2^
GWO	9.413449 × 10^2^	5.770454 × 10^1^	9.182571 × 10^2^	2.268005 × 10^1^	4.893047 × 10^3^	2.336684 × 10^3^
IWOA	1.168813 × 10^3^	7.146982 × 10^1^	9.878765 × 10^2^	4.242970 × 10^1^	6.804606 × 10^3^	2.194565 × 10^3^
ACWOA	1.242042 × 10^3^	5.769592 × 10^1^	1.012184 × 10^3^	2.225332 × 10^1^	7.179991 × 10^3^	7.711065 × 10^2^
BMWOA	1.206188 × 10^3^	1.025870 × 10^2^	1.001529 × 10^3^	2.617066 × 10^1^	7.423863 × 10^3^	1.461209 × 10^3^
CCMWOA	1.272343 × 10^3^	8.580786 × 10^1^	1.046359 × 10^3^	3.441607 × 10^1^	7.758887 × 10^3^	1.121312 × 10^3^
MWOA	2.134655 × 10^3^	3.033994 × 10^2^	1.268728 × 10^3^	4.634955 × 10^1^	2.786309 × 10^4^	8.105906 × 10^3^
ALCPSO	8.553274 × 10^2^	3.411211 × 10^1^	9.049990 × 10^2^	2.715496 × 10^1^	2.134835 × 10^3^	1.176897 × 10^3^
	F10		F11		F12	
	Avg	Std	Avg	Std	Avg	Std
CSAWOA	5.396344 × 10^3^	2.018819 × 10^2^	1.205481 × 10^3^	2.427059 × 10^1^	1.278305 × 10^5^	1.262572 × 10^5^
WOA	5.969759 × 10^3^	7.951794 × 10^2^	1.462925 × 10^3^	9.190777 × 10^1^	3.903354 × 10^7^	3.315709 × 10^7^
PSO	6.122377 × 10^3^	6.208126 × 10^2^	1.284083 × 10^3^	4.368924 × 10^1^	2.853112 × 10^7^	1.387894 × 10^7^
ACO	4.229516 × 10^3^	1.901754 × 10^3^	1.310172 × 10^3^	8.410378 × 10^1^	5.440085 × 10^5^	1.788373 × 10^6^
MFO	5.570030 × 10^3^	9.726606 × 10^2^	7.161747 × 10^3^	8.273801 × 10^3^	1.835168 × 10^8^	2.453205 × 10^8^
HHO	5.634744 × 10^3^	5.617481 × 10^2^	1.258638 × 10^3^	5.005234 × 10^1^	9.708423 × 10^6^	7.270001 × 10^6^
GWO	4.256660 × 10^3^	7.535073 × 10^2^	5.003768 × 10^3^	1.871183 × 10^3^	1.013890 × 10^9^	1.426574 × 10^9^
IWOA	5.404003 × 10^3^	7.112802 × 10^2^	1.349443 × 10^3^	6.956005 × 10^1^	1.098093 × 10^7^	9.252692 × 10^6^
ACWOA	6.376531 × 10^3^	8.273857 × 10^2^	3.124630 × 10^3^	8.313852 × 10^2^	5.355052 × 10^8^	3.558847 × 10^8^
BMWOA	7.433242 × 10^3^	6.742043 × 10^2^	1.685398 × 10^3^	2.007222 × 10^2^	7.174354 × 10^7^	4.288659 × 10^7^
CCMWOA	6.957435 × 10^3^	5.313647 × 10^2^	3.393131 × 10^3^	7.954859 × 10^2^	1.986802 × 10^9^	1.834293 × 10^9^
MWOA	1.016097 × 10^4^	4.323507 × 10^2^	3.392529 × 10^4^	2.161236 × 10^4^	1.676649 × 10^10^	4.762251 × 10^9^
ALCPSO	4.146452 × 10^3^	6.367127 × 10^2^	1.249970 × 10^3^	6.432235 × 10^1^	4.425997 × 10^5^	6.920053 × 10^5^
	F13		F14		F15	
	Avg	Std	Avg	Std	Avg	Std
CSAWOA	1.087455 × 10^4^	6.830157 × 10^3^	2.993102 × 10^3^	1.500653 × 10^3^	3.686163 × 10^3^	2.447275 × 10^3^
WOA	1.514419 × 10^5^	1.093677 × 10^5^	8.480312 × 10^5^	1.106847 × 10^6^	7.932692 × 10^4^	5.394197 × 10^4^
PSO	4.436658 × 10^6^	1.315802 × 10^6^	9.028956 × 10^3^	5.687323 × 10^3^	4.866285 × 10^5^	2.062177 × 10^5^
ACO	2.471514 × 10^4^	2.315307 × 10^4^	3.542310 × 10^4^	3.912741 × 10^4^	2.250046 × 10^4^	1.485083 × 10^4^
MFO	1.043797 × 10^7^	2.452376 × 10^7^	1.692989 × 10^5^	3.279106 × 10^5^	5.711375 × 10^4^	6.359895 × 10^4^
HHO	3.823390 × 10^5^	4.143681 × 10^5^	5.259759 × 10^4^	6.192780 × 10^4^	6.075898 × 10^4^	3.041389 × 10^4^
GWO	2.916017 × 10^8^	8.905218 × 10^8^	4.788951 × 10^5^	5.879218 × 10^5^	1.045465 × 10^7^	5.363672 × 10^7^
IWOA	3.146219 × 10^4^	1.999765 × 10^4^	4.184093 × 10^5^	4.474061 × 10^5^	1.628384 × 10^4^	1.285090 × 10^4^
ACWOA	6.022526 × 10^7^	1.072656 × 10^8^	1.167802 × 10^6^	7.727300 × 10^5^	7.117511 × 10^6^	6.721782 × 10^6^
BMWOA	2.608880 × 10^5^	1.628550 × 10^5^	5.869214 × 10^5^	6.037371 × 10^5^	7.561860 × 10^4^	3.705147 × 10^4^
CCMWOA	1.676398 × 10^8^	2.985046 × 10^8^	1.662225 × 10^6^	1.286575 × 10^6^	5.702362 × 10^6^	9.977447 × 10^6^
MWOA	1.008324 × 10^10^	6.489485 × 10^9^	2.778547 × 10^7^	3.708955 × 10^7^	3.182857 × 10^9^	2.044280 × 10^9^
ALCPSO	1.784766 × 10^4^	1.830509 × 10^4^	1.828320 × 10^4^	2.669947 × 10^4^	1.642649 × 10^4^	1.179018 × 10^4^
	F16		F17		F18	
	Avg	Std	Avg	Std	Avg	Std
CSAWOA	1.993431 × 10^3^	2.046173 × 10^2^	1.879795 × 10^3^	5.416849 × 10^1^	4.469560 × 10^4^	1.485470 × 10^4^
WOA	3.576186 × 10^3^	5.492019 × 10^2^	2.480860 × 10^3^	2.812389 × 10^2^	2.590840 × 10^6^	2.841625 × 10^6^
PSO	2.874169 × 10^3^	2.849564 × 10^2^	2.333822 × 10^3^	2.712448 × 10^2^	1.868239 × 10^5^	1.158438 × 10^5^
ACO	2.255096 × 10^3^	3.530487 × 10^2^	2.116524 × 10^3^	1.867907 × 10^2^	5.652673 × 10^5^	9.855452 × 10^5^
MFO	3.092790 × 10^3^	3.990168 × 10^2^	2.569830 × 10^3^	2.568518 × 10^2^	1.142043 × 10^6^	1.972511 × 10^6^
HHO	3.326637 × 10^3^	4.832132 × 10^2^	2.482845 × 10^3^	3.269189 × 10^2^	9.914022 × 10^5^	9.121447 × 10^5^
GWO	2.696606 × 10^3^	2.778829 × 10^2^	2.300012 × 10^3^	2.640451 × 10^2^	4.976982 × 10^6^	8.027808 × 10^6^
IWOA	3.015057 × 10^3^	3.576796 × 10^2^	2.414047 × 10^3^	2.597844 × 10^2^	2.526347 × 10^6^	2.272492 × 10^6^
ACWOA	3.795578 × 10^3^	4.027167 × 10^2^	2.549285 × 10^3^	2.752048 × 10^2^	2.855844 × 10^6^	3.823050 × 10^6^
BMWOA	3.512041 × 10^3^	4.535979 × 10^2^	2.466857 × 10^3^	2.924330 × 10^2^	3.592779 × 10^6^	3.181471 × 10^6^
CCMWOA	4.154967 × 10^3^	7.219953 × 10^2^	2.741203 × 10^3^	3.119748 × 10^2^	1.076400 × 10^7^	1.132094 × 10^7^
MWOA	7.028317 × 10^3^	1.736420 × 10^3^	1.200415 × 10^4^	1.798172 × 10^4^	2.362614 × 10^8^	2.415703 × 10^8^
ALCPSO	2.601622 × 10^3^	2.980641 × 10^2^	2.151242 × 10^3^	2.137135 × 10^2^	1.789686 × 10^5^	2.280910 × 10^5^
	F19		F20		F21	
	Avg	Std	Avg	Std	Avg	Std
CSAWOA	5.133423 × 10^3^	2.819361 × 10^3^	2.335035 × 10^3^	1.030636 × 10^2^	2.330561 × 10^3^	7.708139
WOA	2.876664 × 10^6^	2.177381 × 10^6^	2.721270 × 10^3^	2.192202 × 10^2^	2.575858 × 10^3^	5.639560 × 10^1^
PSO	1.399142 × 10^6^	6.754508 × 10^5^	2.660120 × 10^3^	2.093334 × 10^2^	2.541418 × 10^3^	3.367995 × 10^1^
ACO	1.953266 × 10^4^	1.680800 × 10^4^	2.325810 × 10^3^	2.149887 × 10^2^	2.407649 × 10^3^	6.110993 × 10^1^
MFO	2.072155 × 10^7^	4.884667 × 10^7^	2.715089 × 10^3^	2.412209 × 10^2^	2.496734 × 10^3^	4.526208 × 10^1^
HHO	3.099739 × 10^5^	1.957465 × 10^5^	2.748788 × 10^3^	2.003736 × 10^2^	2.553659 × 10^3^	3.951806 × 10^1^
GWO	4.123248 × 10^7^	1.417644 × 10^8^	2.571275 × 10^3^	2.327826 × 10^2^	2.434249 × 10^3^	3.108582 × 10^1^
IWOA	3.025580 × 10^4^	5.019168 × 10^4^	2.713724 × 10^3^	1.825884 × 10^2^	2.534867 × 10^3^	5.427024 × 10^1^
ACWOA	1.139322 × 10^7^	2.151953 × 10^7^	2.661944 × 10^3^	1.404524 × 10^2^	2.582430 × 10^3^	3.847761 × 10^1^
BMWOA	7.986303 × 10^5^	8.905633 × 10^5^	2.731562 × 10^3^	1.750996 × 10^2^	2.511681 × 10^3^	6.367424 × 10^1^
CCMWOA	4.111528 × 10^6^	3.950669 × 10^6^	2.699225 × 10^3^	2.437391 × 10^2^	2.623997 × 10^3^	5.188425 × 10^1^
MWOA	3.428632 × 10^9^	2.286595 × 10^9^	3.613992 × 10^3^	2.287634 × 10^2^	2.810297 × 10^3^	7.020447 × 10^1^
ALCPSO	1.377872 × 10^4^	1.453736 × 10^4^	2.346829 × 10^3^	1.565070 × 10^2^	2.421256 × 10^3^	3.445709 × 10^1^
	F22		F23		F24	
	Avg	Std	Avg	Std	Avg	Std
CSAWOA	2.300113 × 10^3^	3.626026 × 10^−1^	2.738885 × 10^3^	6.710687 × 10^1^	2.849177 × 10^3^	8.083320
WOA	7.431238 × 10^3^	1.509092 × 10^3^	3.024497 × 10^3^	1.107738 × 10^2^	3.169132 × 10^3^	8.038509 × 10^1^
PSO	4.827454 × 10^3^	2.729477 × 10^3^	3.125468 × 10^3^	1.389848 × 10^2^	3.171927 × 10^3^	7.133822 × 10^1^
ACO	5.181166 × 10^3^	2.021525 × 10^3^	2.734021 × 10^3^	4.492808 × 10^1^	2.945934 × 10^3^	6.350869 × 10^1^
MFO	6.740986 × 10^3^	1.076680 × 10^3^	2.826454 × 10^3^	3.314846 × 10^1^	2.992839 × 10^3^	2.822412 × 10^1^
HHO	6.696083 × 10^3^	1.613072 × 10^3^	3.108197 × 10^3^	8.660652 × 10^1^	3.423638 × 10^3^	1.248917 × 10^2^
GWO	5.728036 × 10^3^	1.181956 × 10^3^	2.928817 × 10^3^	6.217652 × 10^1^	3.088805 × 10^3^	5.325270 × 10^1^
IWOA	5.413263 × 10^3^	2.315658 × 10^3^	2.994173 × 10^3^	8.463477 × 10^1^	3.169425 × 10^3^	8.861861 × 10^1^
ACWOA	5.520807 × 10^3^	2.193569 × 10^3^	3.051088 × 10^3^	8.093732 × 10^1^	3.188050 × 10^3^	6.403894 × 10^1^
BMWOA	5.065750 × 10^3^	3.251119 × 10^3^	2.946540 × 10^3^	7.441813 × 10^1^	3.097204 × 10^3^	7.241855 × 10^1^
CCMWOA	7.263658 × 10^3^	1.487237 × 10^3^	3.139778 × 10^3^	1.184377 × 10^2^	3.338432 × 10^3^	9.724981 × 10^1^
MWOA	1.137426 × 10^4^	8.477184 × 10^2^	3.569427 × 10^3^	1.497377 × 10^2^	3.763464 × 10^3^	1.366447 × 10^2^
ALCPSO	5.007939 × 10^3^	1.573927 × 10^3^	2.793884 × 10^3^	4.937573 × 10^1^	2.997061 × 10^3^	4.894870 × 10^1^
	F25		F26		F27	
	Avg	Std	Avg	Std	Avg	Std
CSAWOA	2.878809 × 10^3^	1.636850	2.886667 × 10^3^	3.457459 × 10^1^	3.201860 × 10^3^	5.477334
WOA	2.946038 × 10^3^	2.909135 × 10^1^	7.680049 × 10^3^	1.224866 × 10^3^	3.374706 × 10^3^	9.551947 × 10^1^
PSO	2.902323 × 10^3^	2.556995 × 10^1^	5.050198 × 10^3^	1.979579 × 10^3^	3.203690 × 10^3^	1.119410 × 10^2^
ACO	2.895989 × 10^3^	1.960615 × 10^1^	4.532015 × 10^3^	3.125279 × 10^2^	3.230319 × 10^3^	1.530899 × 10^1^
MFO	3.276917 × 10^3^	3.301971 × 10^2^	6.022053 × 10^3^	4.783455 × 10^2^	3.255803 × 10^3^	2.456574 × 10^1^
HHO	2.910117 × 10^3^	1.695938 × 10^1^	6.261523 × 10^3^	2.099172 × 10^3^	3.342235 × 10^3^	7.058319 × 10^1^
GWO	3.227474 × 10^3^	1.997499 × 10^2^	6.244668 × 10^3^	7.503779 × 10^2^	3.381174 × 10^3^	9.829200 × 10^1^
IWOA	2.929780 × 10^3^	2.473938 × 10^1^	6.761334 × 10^3^	1.114673 × 10^3^	3.301062 × 10^3^	6.713109 × 10^1^
ACWOA	3.174852 × 10^3^	1.284224 × 10^2^	7.321649 × 10^3^	1.359854 × 10^3^	3.458949 × 10^3^	1.516728 × 10^2^
BMWOA	3.014063 × 10^3^	4.075813 × 10^1^	6.777090 × 10^3^	1.156228 × 10^3^	3.318944 × 10^3^	7.029540 × 10^1^
CCMWOA	3.339831 × 10^3^	1.191777 × 10^2^	8.887845 × 10^3^	7.478576 × 10^2^	3.575975 × 10^3^	1.416473 × 10^2^
MWOA	8.594095 × 10^3^	2.001827 × 10^3^	1.269589 × 10^4^	1.319486 × 10^3^	4.614543 × 10^3^	4.734169 × 10^2^
ALCPSO	2.897906 × 10^3^	1.706257 × 10^1^	4.866300 × 10^3^	6.179493 × 10^2^	3.262451 × 10^3^	3.112561 × 10^1^
	F28		F29		F30	
	Avg	Std	Avg	Std	Avg	Std
CSAWOA	3.208055 × 10^3^	2.992108 × 10^1^	3.573434 × 10^3^	1.208427 × 10^2^	2.337569 × 10^4^	1.879750 × 10^4^
WOA	3.304798 × 10^3^	3.006682 × 10^1^	4.764294 × 10^3^	4.148598 × 10^2^	1.050050 × 10^7^	9.531813 × 10^6^
PSO	3.253278 × 10^3^	2.354970 × 10^1^	4.264288 × 10^3^	2.332446 × 10^2^	3.935993 × 10^6^	1.913860 × 10^6^
ACO	3.292877 × 10^3^	9.808876 × 10^1^	3.743716 × 10^3^	2.014103 × 10^2^	1.443995 × 10^4^	7.306818 × 10^3^
MFO	4.534127 × 10^3^	1.039251 × 10^3^	4.128816 × 10^3^	2.917697 × 10^2^	5.808740 × 10^5^	8.368010 × 10^5^
HHO	3.255874 × 10^3^	3.160452 × 10^1^	4.488520 × 10^3^	4.397915 × 10^2^	1.720157 × 10^6^	1.161780 × 10^6^
GWO	4.109921 × 10^3^	5.163692 × 10^2^	4.227999 × 10^3^	2.331320 × 10^2^	2.862588 × 10^7^	1.171162 × 10^8^
IWOA	3.271534 × 10^3^	2.432468 × 10^1^	4.244064 × 10^3^	2.644432 × 10^2^	5.182240 × 10^5^	3.387571 × 10^5^
ACWOA	3.817232 × 10^3^	2.245604 × 10^2^	4.664105 × 10^3^	3.669519 × 10^2^	7.285437 × 10^7^	5.810248 × 10^7^
BMWOA	3.407279 × 10^3^	5.076257 × 10^1^	4.826111 × 10^3^	4.375699 × 10^2^	6.158149 × 10^6^	4.291418 × 10^6^
CCMWOA	4.590795 × 10^3^	4.186615 × 10^2^	5.140391 × 10^3^	4.840076 × 10^2^	7.551451 × 10^7^	5.870687 × 10^7^
MWOA	9.720803 × 10^3^	1.887081 × 10^3^	1.095361 × 10^4^	6.484789 × 10^3^	2.590888 × 10^9^	2.159377 × 10^9^
ALCPSO	3.242643 × 10^3^	4.270598 × 10^1^	3.895661 × 10^3^	2.421831 × 10^2^	1.597415 × 10^4^	9.506606 × 10^3^

**Table 10:** The accuracy of each model and the corresponding learning rate after five rounds of cross-validation.

Model		AVG	STD	Fold 1	Fold 2	Fold 3	Fold 4	Fold 5
YOLOv8	Best LR	0.004	0.002	0.003	0.001	0.006	0.004	0.007
	Best mAP50	0.766	0.013	0.749	0.781	0.758	0.769	0.773
	Worst LR	0.086	0.011	0.07	0.09	0.1	0.08	0.09
	Worst mAP50	0.517	0.021	0.516	0.538	0.487	0.510	0.535
YOLOv11	Best LR	0.005	0.003	0.005	0.004	0.002	0.003	0.01
	Best mAP50	0.721	0.010	0.725	0.719	0.720	0.706	0.734
	Worst LR	0.20	0.28	0.06	0.04	0.08	0.7	0.1
	Worst mAP50	0.62	0.033	0.631	0.583	0.662	0.587	0.630
YOLOv9	Best LR	0.003	0.001	0.002	0.005	0.003	0.002	0.004
	Best mAP50	0.702	0.013	0.708	0.691	0.724	0.696	0.695
	Worst LR	0.076	0.021	0.08	0.06	0.05	0.1	0.09
	Worst mAP50	0.456	0.066	0.481	0.410	0.373	0.541	0.477
YOLOV10	Best LR	0.003	0.001	0.001	0.003	0.004	0.005	0.002
	Best mAP50	0.685	0.015	0.687	0.701	0.673	0.666	0.698
	Worst LR	0.04	0.016	0.02	0.05	0.06	0.04	0.03
	Worst mAP50	0.399	0.055	0.481	0.362	0.393	0.416	0.339
YOLOv7-tiny	Best LR	0.004	0.002	0.001	0.004	0.005	0.004	0.006
	Best mAP50	0.669	0.015	0.694	0.669	0.658	0.660	0.662
	Worst LR	0.08	0.016	0.08	0.06	0.07	0.1	0.09
	Worst mAP50	0.452	0.087	0.379	0.481	0.505	0.344	0.551

**Table 11:** Comparison of the outcomes of several methods for object detection.

Models	P (%)	mAP50 (%)	Recall (%)	FLOPs/G	Parameters	FPS
YOLOv7-tiny [6]	67.3	66.5	68.0	13.2	6.0 × 10^6^	38
YOLOv8n	71.2	70.1	63.8	14.3	3.35 × 10^6^	67
YOLOv9 [7]	70.0	69.4	63.1	21.8	6.54 × 10^6^	47
YOLOv10n [8]	69.5	67.9	64.2	12.7	3.19 × 10^6^	68
YOLOv11n [9]	73.4	72.7	70.7	9.6	2.82 × 10^6^	63
CSAWOA-YOLOv8	76.1	76.2	72.1	18.2	3.87 × 10^6^	65

**Table 12:** Comparison of the outcomes of several methods for object detection.

Class	Baseline YOLOv8			CSAWOA-YOLOv8 (Ours)		
	P (%)	R (%)	mAP50 (%)	P (%)	R (%)	mAP50 (%)
Person	82.0	78.0	83.5	84.5	82.0	86.5
Chair	78.0	72.0	78.0	80.5	76.0	81.0
Trash bin	72.0	64.0	70.5	77.5	72.0	75.5
Vehicle or cart	68.0	58.0	66.0	74.0	68.0	72.5
Handbag	63.0	55.0	62.5	70.5	67.0	72.0
Fire hydrant	64.0	56.0	60.1	69.5	68.0	69.7
All	71.2	63.8	70.1	76.1	72.1	76.2

**Table 13:** Results after adding different module and methods.

Number	YOLOv8	T-CBS	BiFormer	CSAWOA	Improved Focal Loss	P (%)	R (%)	mAP50 (%)	mAP50-90 (%)
1	√					71.2	63.8	70.3	49.1
2	√	√				73.0	65.2	72.2	52.6
3	√		√			71.8	67.2	71.0	50.9
4	√			√		71.4	62.7	72.7	51.1
5	√	√		√		73.8	64.4	74.1	52.4
6	√		√	√		74.6	67.8	75.5	53.5
7	√	√	√			75.1	68.9	74.7	55.9
8	√				√	71.6	63.2	71.9	51.6
9	√	√			√	73.4	65.3	72.6	52.3
10	√		√		√	72.3	65.1	73.1	52.9
11	√			√	√	73.2	64.5	73.5	54.1
12	√	√	√	√	√	76.1	72.1	76.2	57.6

**Table 14:** Verifying the generalization ability of a model.

Model	P (%)	mAP50 (%)	Recall (%)	FPS
YOLOv8	79.51 ± 0.42	73.18 ± 0.38	64.72 ± 0.51	33
YOLOv9	78.40 ± 0.45	69.75 ± 0.41	62.51 ± 0.53	35
YOLOv10	74.42 ± 0.52	64.31 ± 0.47	61.55 ± 0.58	34
YOLOv11	85.36 ± 0.35	76.08 ± 0.32	70.96 ± 0.46	38
UR-YOLO	86.74 ± 0.31	75.91 ± 0.36	64.05 ± 0.49	32
Improved CSAWOA YOLOv8	87.05 ± 0.28	77.24 ± 0.31	72.18 ± 0.43	35

## Data Availability

Data will be made available on request.
